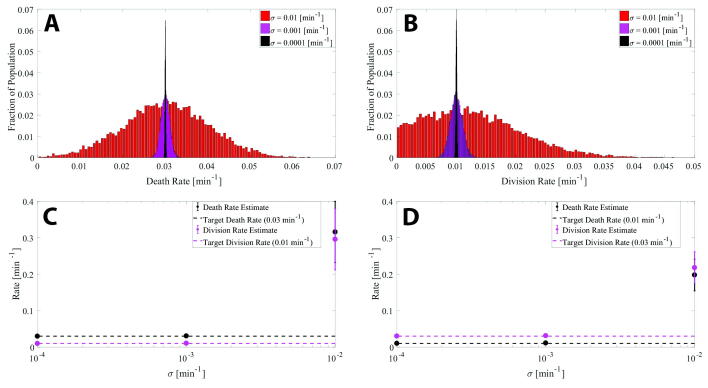# RESTAMP – Rate estimates by sequence-tag analysis of microbial populations

**DOI:** 10.1016/j.csbj.2021.01.017

**Published:** 2021-01-19

**Authors:** Anel Mahmutovic, Aaron Nicholas Gillman, Silje Lauksund, Natasha-Anne Robson Moe, Aime Manzi, Merete Storflor, Pia Abel zur Wiesch, Sören Abel

**Affiliations:** a Department of Pharmacy, Faculty of Health Sciences, UiT – The Arctic University of Norway, 9037 Tromsø, Norway; b Department of Veterinary and Biomedical Sciences, The Pennsylvania State University, PA 16802, USA; c Centre for Molecular Medicine Norway, Nordic EMBL Partnership, 0318 Oslo, Norway; d Department of Biology, The Pennsylvania State University, University Park, PA 16802, USA; e Huck Institutes of the Life Sciences, The Pennsylvania State University, University Park, PA 16802, USA

**Keywords:** Stochastic population dynamics, Multinomial random sampling, Bottleneck, Division rate, Death rate, Founder population size

## Abstract

Microbial division rates determine the speed of mutation accumulation and thus the emergence of antimicrobial resistance. Microbial death rates are affected by antibiotic action and the immune system. Therefore, measuring these rates has advanced our understanding of host-pathogen interactions and antibiotic action. Several methods based on marker-loss or few inheritable neutral markers exist that allow estimating microbial division and death rates, each of which has advantages and limitations. Technical bottlenecks, i.e., experimental sampling events, during the experiment can distort the rate estimates and are typically unaccounted for or require additional calibration experiments.

In this work, we introduce RESTAMP (Rate Estimates by Sequence Tag Analysis of Microbial Populations) as a method for determining bacterial division and death rates. This method uses hundreds of fitness neutral sequence barcodes to measure the rates and account for experimental bottlenecks at the same time. We experimentally validate RESTAMP and compare it to established plasmid loss methods.

We find that RESTAMP has a number of advantages over plasmid loss or previous marker based techniques. (i) It enables to correct the distortion of rate estimates by technical bottlenecks. (ii) Rate estimates are independent of the sequence tag distribution in the starting culture allowing the use of an arbitrary number of tags. (iii) It introduces a bottleneck sensitivity measure that can be used to maximize the accuracy of the experiment.

RESTAMP allows studying microbial population dynamics with great resolution over a wide dynamic range and can thus advance our understanding of host-pathogen interactions or the mechanisms of antibiotic action.

## 1 Introduction

During the last decade, considerable advances have been made toward understanding the detailed population dynamics of pathogens [1], [2], [3], [4], [5], [6]. These were made possible by theoretical [1], [3], [7] and methodological developments such as signature tagging [1], [8] and next-generation sequencing [9]. Mutations mainly occur during replication, and therefore microbial division rates are the main driver of the rates with which pathogens acquire antibiotic resistance or evade vaccines. A complete understanding of the complex population dynamics from the level of individual dynamical processes, such as division and death, offers a way to counter the rise of antibiotic resistance [5], [10], [11] and for rational design of vaccines and therapies against pathogen colonization and infection [1], [2], [3], [4].

One set of methods for determining the division and death rate of a microbial population relies on a single identifiable marker that loses signal strength with each division. These markers include phenotypic tags such as conditionally non-replicative plasmids [12] unstable plasmids [13] and fluorescent inclusion bodies [14]. Plasmid based markers are lost during cell division from inheritance in a single daughter cell or a fraction of cells. Alternatively, fluorescent dye markers are diluted during cell growth and division [15], [16]. The ratio of marker-to-marker-less cells or the magnitude of the marker signal, are used to estimate the division and death rate. These methods yield robust and accurate rate estimates for times short enough to ensure that the markers have not been completely diluted out of the population. For example, Frenoy et al. [12] used conditionally non-replicative plasmids in *Escherichia coli* to show that mutation rates are systematically overestimated in bacteria under stressful conditions unless the individual division and death rates are taken into account. In the study by Myhrvold et al. [14] self-aggregating fluorescent proteins were used to estimate division rates and death rates, which were subsequently used to inform a mathematical model of the population dynamics of *E. coli* in the mouse gut. Although successful, the application of marker-based techniques is dependent on the assumption that the markers are fitness neutral, i.e. do not change the wild-type microbial division rates and death rates. This is typically experimentally challenging to confirm within the context of within-host infection models.

An alternative approach relies on the use of distinguishable inheritable markers, where each marker labels a subpopulation of cells and the change in the composition of the total population is used to infer division and death rates by quantitative stochastic population-dynamic approaches. In principle, these methods offer an unlimited observation time with their accuracy limited by the total number of unique tags. For example, sequence tags inserted into a fitness neutral locus in the genome (wild type isogenic tagged strains; WITS) have been successfully used to investigate the pathogenesis of *Salmonella enterica* serovar Typhimurium [1] to quantify the effects of different vaccines [3] and investigate the colonization of the cecal lymph node [2]. Vlazaki et al. reviews the underlying mathematical approaches for estimating rates and the applications in more detail [15]. However, it remains unclear how the error in the rate estimates depends on the specific experimental protocol. Importantly, the impact of technical population bottlenecks on rate estimates is typically not accounted for and when addressed require additional calibration experiments [3]. Technical bottlenecks can change the population composition and thereby the basis for rate estimates [17]. Bottlenecks are often inevitably introduced during the experiment, for example when sampling a small volume from a large volume or when sequencing a limited number of cells. Moreover, the typical mathematical analysis of WITS data constrains the experimental design, and requires a uniform distribution of tags in the starting culture to work accurately. This is experimentally challenging to achieve for a large number of tags and therefore restricts the number of useable distinguishable sequence tags.

**Fig. 1:**
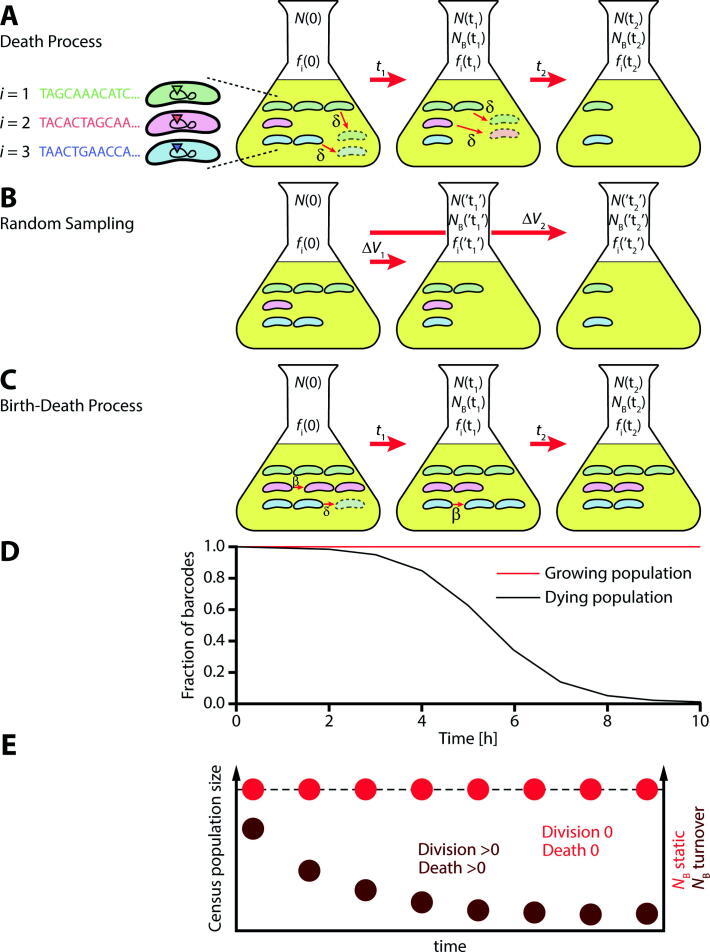
Schematic of the experimental setups. (A) Illustrates a pure death process for a population of bacteria with *i* = 1, 2, 3,…,*k* = 1000 unique 30 base pairs sequence tags at a fitness neutral location in the genome. The three magnified cells illustrate the genome within the bacteria (black circle) with potential fitness neutral locations (rectangles) and the fitness neutral location with a sequence tag (color of rectangle/bacterium). The *i*:th subpopulation is initially present with a frequency *f*_i_(0) where the total population size is *N*(0). The bacteria undergo random death events for a length of time *t*_1_ with rate *δ* per unit time after which the total population size is *N*(t_1_) and the frequency of the *i*:th subpopulation is *f*_i_(t_1_). The founder population size at time *t*_1_, *N*_B_(t_1_), is calculated by comparing the frequency of bacteria at time *t*_1_ with the initial frequency, *f*_i_(0) (equation (1)). After an elapsed time *t*_2_ > *t*_1_ fewer cells remain with *N*(t_2_) < *N*(t_1_) and *N*_B_(t_2_) < *N*_B_(t_1_). (B) Random sampling of the initial population with the aim of emulating a pure death process. The volume ${\Delta v}_{t}$ is sampled from a large volume in which tagged bacteria are suspended such that the number of cells sampled is equal to the number of cells having undergone a death process for a length of time *t* (A). The founder population size, *N*_B_(‘t_1_′), and the population size, *N*(‘t_1_′), are determined in the sample *Δ*v_t_ and are used to estimate the death rate, *δ*, as it would be in a pure death process (see *5.1 – Emulating a death process by sampling*). The random sampling process in itself is not a real time-dependent process, which is indicated by apostrophes around *t*. (B) A birth–death process that includes both death events and division events with rate *δ* per unit time and *β* per unit time, respectively. By measuring the total population size and the genetic drift in terms of the founder population size the division rate and death rate can be estimated. (D) Simulation for tag loss in two populations with the same initial composition but different growth and death rates. The mathematical framework from [21] was adopted to investigate the mean fraction of unique sequence tags (y-axis, RESTAMP) that survives until time *t* (x-axis) in a birth–death process. The black line represents a population that dies more quickly than it replicates. The red line represents a population that replicates more than it dies. The parameters for the simulation are the same as in Figure Supplementary Fig. 1. (E) Schematic representation of the simultaneous change in census population size (black dotted line) and founding population size (red dots) for two different sets of division and death rates (light and dark red, respectively). For both scenarios, the census population size, i.e. the total number of cells remains constant. However, depending on the magnitude of the rates, different profiles for *N*_B_ emerge over time. (For interpretation of the references to color in this figure legend, the reader is referred to the web version of this article.)

A number of studies have employed population genetic concepts to study microbial dynamics qualitatively [8], [18], [19]. The sequence tag-based analysis of microbial populations (STAMP) method allows for an indefinite number of sequence tags to be incorporated, limited only by the throughput of next-generation sequencing. Like WITS, STAMP allows for a much longer observation time than marker-loss methods. STAMP also prescribes a simple way to aggregate the change of each individually tagged subpopulation into a single measure, the founder population size. The founder population size carries a simple interpretation as the size of a population that survived a death event [20] (Fig. 1). These events are also often referred to as bottlenecks and can correspond to host-pathogen interactions, e.g. physical barriers, immune defenses, nutritional limitations, etc. The smaller the size of the founder population, compared to the initial population, the greater the stringency of the bottleneck. This measure can be affected by artificially changing the composition of the tagged population by technical handling of the sample after the biological process, for example by transferring only part of a sample by pipetting or by sequencing to an insufficient sequencing depth. These events can be seen as random sampling events [17].

In this work, we specifically distinguish them as technical bottlenecks in contrast to the biological bottlenecks due to birth–death processes. In combination with the current bacterial burden, measured by colony forming units (CFU), the founder population size can help understand the detailed dynamics of a population. For example, if the current bacterial burden is linked to a small founder population size that would indicate growth of the microbes, while the same bacterial burden linked to a large founder population size would indicate slower growth. Loss of tag diversity indicates bacterial death, and the more bacteria are killed, the more tag diversity is lost. Fig. 1D illustrates how tags are lost depending on the division or death rates. At one extreme, when there is only death, the founder population size decreases in step with the bacterial burden. At the other extreme, when there is only growth, the tag diversity does not change and the founder population size remains constant. Hence, the simultaneous time-course data for the bacterial burden and the founder population size provides a unique signature for how rapidly cells divide and die that has been exploited to qualitatively assess the relative contribution to bacterial division and death events [8] (Fig. 1E). However, to date there exists no mathematical framework for the STAMP method able to infer these rates from CFU and founder population size values.

In this work, we develop and expand the population genetic framework for the STAMP method to quantify the relationship between the founder population size and microbial division and death rates. Our method, RESTAMP (Rate Estimates by Sequence Tag Analysis of Microbial Populations), allows for estimating rates via measurements of the founder population size and colony forming units that are independent of the distribution of the sequence tags. The RESTAMP method relies on estimating the magnitude of the fluctuations in the genetic composition of cells as they undergo random birth–death processes. Unavoidable technical bottlenecks such as sampling a small volume and sequencing influence the fluctuations in the genetic composition and can lead to biased rate estimates. We show that measuring the founder population size permits for a simple analytical scheme to correct for technical bottlenecks. Finally, we propose a bottleneck sensitivity measure and show how the bottleneck sensitivity depends on experimental parameters. This measure can be used to design experiments to maximize the accuracy and precision of bacterial division rate and death rate estimates. The method is validated by simple control experiments, aimed at emulating a pure death process (Fig. 1AB), and for cells growing in lysogeny broth (LB) media (Fig. 1C) by comparison with the well-established marker-loss plasmid segregation (PS) method.

## 2 Results – theory

In this section we develop the population genetic framework for the experimental STAMP method (equation (1)) against the backdrop of a random birth–death process. In subsection 2.1 we consider the ideal case where there is no influence of technical bottlenecks, e.g. due to sequencing or sampling, and derive an equation (equation (5)) that relates the mean founder population size to the division and death rate of a population of cells. In subsection 2.2 we explore the influence of technical bottlenecks on the analysis of sequence tags for estimating division and death rates. Here we also explore the impact of using an experimental estimate of the reference state at time 0 by sampling. The main result of this subsection is equation (7), which prescribes how to subtract the influence of technical bottlenecks. In subsection 2.3 we propose a bottleneck sensitivity measure, the purpose of which is to quantify the sensitivity in the rate estimates against making an error in the bottleneck correction terms in equation (7). The rationale for the bottleneck sensitivity measure is to maximize the variance in the frequencies of sequence tags due to random birth–death events relative to the variance in the frequencies induced by experimental sampling events.

### 2.1 RESTAMP – bacterial division rate and death rate estimates from the founder population size

To model the population dynamics of *k* (see Table 1 for the definition and meaning of variables) distinguishable and independent subpopulations of cells we adopt the standard stochastic framework for which the trajectories are assumed to be continuous-time Markov processes [22].

**Table 1:** Summary of variables used in this work.

Variable	Meaning	Comments
*Section 2.1: RESTAMP – Bacterial division rate and death rate estimates from the founder population size*		
*k*	Total number of subpopulations.	For the experiments in this work, *k* = 1000 and corresponds to the total number of unique 30 base pairs sequence tags.
*i*	An index denoting a specific subpopulation of cells.	The range is *i* = 1,2,…,*k*.
*β*	Division rate.	Defined as the inverse average time it takes for a cell to divide.
*δ*	Death rate.	Defined as the inverse average time it takes for a cell to die.
*r*	Net growth rate.	The net growth rate is defined as the difference between the division rate *β* and the death rate *δ, r = β- δ.*
*t*	The length of time cells undergo a birth–death process.	
<*n_i_(t)* > a, b	Average number of cells with a sequence tag insertion *i* at time *t*.	
<*N(t)*>	Total average number of cells at time *t*.	
*f*_i_(*t*)	The proportion of cells having undergone a birth–death process for a length of time *t* with a sequence tag *i*.	<*f_i_(t)* > denotes the average proportion of cells where the average is with respect to realizations. *f_i_*(0) is the proportion of cells with sequence tag *i* in the inoculum.
*Var(f*_i_(*t*))	The variance in the proportion of cells having undergone a birth–death process for a length of time *t* with a sequence tag insertion at site *i*.	The variance is with respect to repetitions of the experiment.
*f_i_*	The proportion of cells having undergone a random sampling event with a sequence tag insertion at site *i*.	In this work we assume that experimental samplings (technical bottlenecks), e.g. due to pipetting or sequencing, are modeled as random sampling processes, whereby a subset of cells are sampled such that each cell has the same chance of ending up in the sample.
*Var(f*_i_)	The variance in the proportion of cells having undergone a random sampling event with a sequence tag insertion at site *i*.	
*N_B_(t)*	The founder population size is calculated by comparing the proportion of cells with tag *i* at time *t* with the proportion of cells with tag *i* in the inoculum (equation (1)).	The magnitude of *N*_B_(*t*) signifies the biological bottleneck, where a small *N*_B_(*t*) corresponds to a stringent bottleneck and vice versa. The explicit time-dependence signifies that the founder population size is calculated for cells having undergone a birth–death process. In contrast, *N*_B_ (without time dependence) refers to a technical bottleneck.
*Section 2.2: RESTAMP – Correcting for unwanted random sampling events (bottlenecks)*		
*m_S_*	The number of bottlenecks that the sample at time *t* undergoes.	The typical value is *m_S_* = 2 in our experiments which include an experimental sampling bottleneck and a sequencing bottleneck. For the controlled death experiments, *m_S_* = 1.
*m_I_*	The number of bottlenecks that the sample at time 0 undergoes.	The typical value is *m_I_* = 2 in our experiments which include an experimental sampling bottleneck and a sequencing bottleneck. For the controlled death experiments, *m_I_* = 1.
*j*	A specific random sampling event.	The range is *j* = 1,2,…,*m_I_* for the sample at time 0 and *j* = 1,2,…,*m_S_* for the sample at time *t.*
*S_j_*	The sample size of the *j*:th random sampling event.	
*I_j_*	The inoculum size of the *j*:th random sampling event.	
*Section 2.3: RESTAMP – Bottleneck sensitivity of bacterial division and death rate estimates*		
*N_B_*	The sample size in a random sampling event.	*N*_B_ is the notation used for the magnitude of a technical bottleneck e.g. the sample size in the transferred volume when pipetting or the sequencing depth when loading the sample on a sequencing chip.
*s_B_(t)*	Bottleneck sensitivity.	Defined as the ratio of the variance in the subpopulation proportions due to a random sampling event and a birth–death process, i.e. $s_{B}\left(t\right)={\mathrm{Var}}_{B}\left(f_{i}\right)/{\mathrm{Var}}_{\mathrm{BD}}\left(f_{i}\left(t\right)\right)$. The purpose of *S_B_(t)* is to serve as a measure of how sensitive division rate and death rate estimates are to technical bottlenecks.
*Section 5.1: Materials and Methods – Emulating a death process by sampling*		
*Δv_t_*	The size of the sampled volume.	This is the volume sampled to emulate a death process at time *t* where *Δv_t_ = Δv_0_e^-δt^* with *Δv_0_* being the sampled volume of the starting culture at *t* = 0 (*Δv_0_* = 1 ml in our experiments).
*s*	The number of cells at time *t* for a death process or the number of cells in the sample *Δv_t_*.	
*P(n_i_ = s)*	The probability of sampling *s* cells with a sequence tag insertion at site *i* in the sample *Δv_t_*.	
*P(n_i_(t) = s)*	The probability of *s* cells remaining at time *t* in a pure death process.	
*p*	Probability of a single cell surviving until time *t*.	*p = e^-δt^*
*Section 5.2: Materials and Methods – Plasmid Segregation (PS)*		
*F(t)*	Proportion of cells with a plasmid at time *t*.	

a All cell numbers are implicitly expressed as per unit volume.

b Averages over repetitions are denoted with angular brackets <>.

We consider a birth–death process where both the time until the next event and the type of event are random variables. The event is either a division with rate *β* per unit time, defined as the inverse average time it takes for a cell to divide, or a death event with rate *δ* per unit time, defined as the inverse average time it takes for a cell to die. The division rate and the death rate do not depend on the specific sequence tag *i* = 1, 2, 3,…,*k* since the insertion is fitness neutral. Hence, each subpopulation *i* undergoes random division and death events with the same rates for a length of time *t* resulting in a random subpopulation size, *n_i_(t)*. Consequently, the proportion of cells with a sequence tag *i*, *f_i_(t)*, is also a random variable where $f_{i}\left(t\right)=n_{i}\left(t\right)/N\left(t\right)$ and $N\left(t\right)=\sum_{i=1}^{k}n_{i}\left(t\right)$ is the total population size at time *t*. The implication is that the founder population size, *N_B_(t)*, as determined by equation (1), is also to be treated as a random variable.

(1) $$N_{B}\left(t\right)=\frac{1}{\frac{1}{k}\sum_{i=1}^{k}\frac{{\left(f_{i}\left(t\right)-f_{i}\left(0\right)\right)}^{2}}{f_{i}\left(0\right)\left(1-f_{i}\left(0\right)\right)}}$$

The equation for the founder population size is derived in the context of a multinomial random-sampling process, where it is interpreted as the population size that survived a multinomial random sampling event [20]. The equation was originally derived by [8] for diploid organisms and adapted by [20] for haploid organisms. The validity of this interpretation is contingent on a small volume being sampled so that the proportion of subpopulation *i* before sampling, *f_i_(0)*, remains unchanged after sampling, *f_i_*. Here we introduce the notation *f_i_* without an explicit time dependence to signify that the change in subpopulation proportions are due to a random sampling process (technical bottleneck) in contrast to changes in subpopulation proportions due to a birth–death process (biological bottleneck) (see Table 1). In this section, we focus on the stochastic birth–death process where we seek to relate the founder population size to the division rate and death rate. By taking the inverse of equation (1) and apply the mean operator, <>, we get

(2) $$<{N_{B}\left(t\right)}^{-1}>=\frac{1}{k}\sum_{i=1}^{k}\frac{{<\left(f_{i}\left(t\right)-f_{i}\left(0\right)\right)}^{2}>}{f_{i}\left(0\right)\left(1-f_{i}\left(0\right)\right)}$$

where the mean is with respect to repeating the birth–death processes given an initial proportion, *f_i_(0)*. This assumes that the initial tag frequencies can be determined precisely. Next, we assume that the total population size is large enough to make the error in the approximation of the mean subpopulation proportion as ${<f}_{i}\left(t\right)>\approx \frac{{<n}_{i}\left(t\right)>}{<N\left(t\right)>}$ negligible. Since the mean subpopulation size for a birth–death process is given by $<n_{i}\left(t\right)>=n_{i}\left(0\right)e^{\left(\beta -\delta \right)t}$ and the total population size is given by $<N\left(t\right)>=N\left(0\right)e^{\left(\beta -\delta \right)t}$, it follows that ${<f}_{i}\left(t\right)>\approx f_{i}\left(0\right)=n_{i}\left(0\right)/N\left(0\right)$. Substituting ${<f}_{i}\left(t\right)>\approx f_{i}\left(0\right)$ in the numerator under the sum in equation (2) we get

(3) $$<{N_{B}\left(t\right)}^{-1}>\approx \frac{1}{k}\sum_{i=1}^{k}\frac{\mathrm{Var}(f_{i}\left(t\right))}{f_{i}\left(0\right)\left(1-f_{i}\left(0\right)\right)}$$

where by definition, $Var\left(f_{i}\left(t\right)\right)={<\left(f_{i}\left(t\right)-{<f}_{i}\left(t\right)>\right)}^{2}>$ is the variance in the subpopulation proportions. Equation (3) is likewise valid for a multinomial random sampling process for which the mean subpopulation size after sampling *N_B_* cells is $<n_{i}>=N_{B}f_{i}\left(0\right)$ and $<f_{i}>=<n_{i}>/N_{B}=f_{i}\left(0\right)$. Using the error propagation method we derive (see *5.4 - The variance in the proportion of cells with respect to repetitions for a birth*–*death process*) the variance in the subpopulation proportions for a birth–death process which reads

(4) $$Var\left(f_{i}\left(t\right)\right)\approx \frac{\left(\beta +\delta \right)\left(1-e^{-\left(\beta -\delta \right)t}\right)}{\left(\beta -\delta \right)N\left(0\right)}f_{i}\left(0\right)\left(1-f_{i}\left(0\right)\right)$$

Substituting equation (4) in (3) and making the approximation $<N_{B}\left(t\right)>\approx 1/<{N_{B}\left(t\right)}^{-1}>$ (i.e. the average of the inverse is not equal to the inverse of the average) we get

(5) $$<N_{B}\left(t\right)>\approx \frac{\left(\beta -\delta )N(0\right)}{\left(\beta +\delta \right)\left(1-e^{-\left(\beta -\delta \right)t}\right)}$$

Equation (5) shows that the mean founder population size due to a birth–death process is independent of the distribution of tags. This simplifies the work of an experimenter aiming to estimate bacterial division rates and death rates as care need not be taken to produce a library of cells with a specific distribution of sequence tags. Consequently, it becomes a simple matter to analyze an arbitrary number of sequence tags which are all aggregated into the founder population size. We also note that < *N_B_(t)* > is directly proportional to the total population size at *t* = 0, *N(0)*, and is given in units of per volume. This implies that the mean founder population size must be expressed with respect to the same unit of volume for equation (5) to be dimensionally consistent. For example, if *N(0)* is determined as the CFU per ml and only 200 µl is sampled for determining *N_B_*, then the measured *N_B_* values need to be multiplied by 5 (200 µl × 5 = 1 ml). Using equation (5) and the exponential growth model for which $<N\left(t\right)>=N\left(0\right)e^{\left(\beta -\delta \right)t}$, we solve for the division rate and death rate to get

(6ab) $$\left\{\begin{array}{c} \delta \approx \frac{r}{2}\left[\frac{N(0)}{<N_{B}\left(t\right)>\left(1-\frac{N(0)}{<N\left(t\right)>}\right)}-1\right]\\ \beta \approx r+\delta \end{array}\right)$$

where *r* is the net growth rate and can be experimentally estimated as the slope of a regression line of ln(CFU) versus time.

### 2.2 RESTAMP – Correcting for technical bottlenecks

**Fig. 2:**
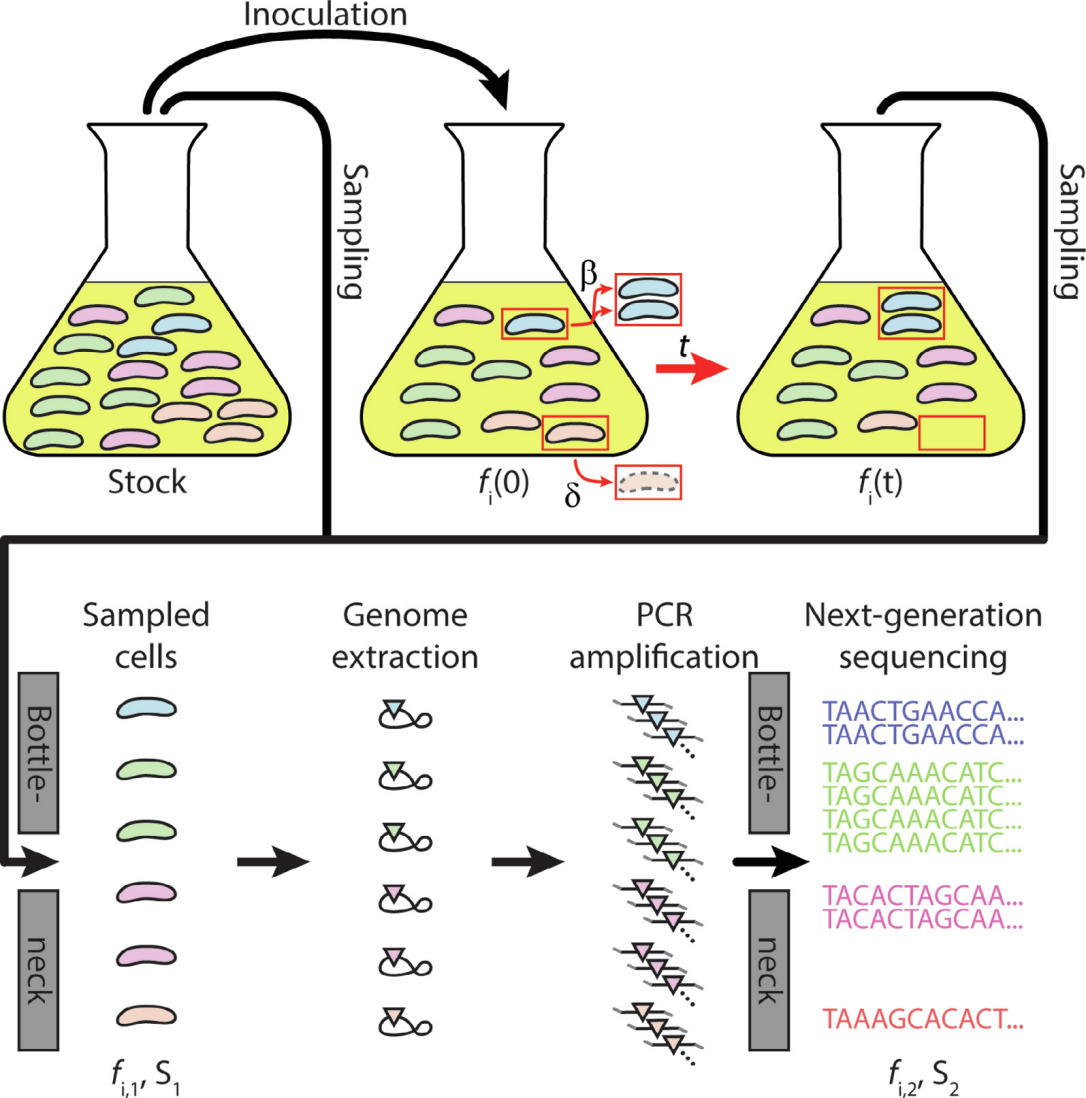
The bottlenecks in a typical experimental setup for RESTAMP rate estimates. The schematic lays out a typical experimental setup to determine division and death rates of cells by RESTAMP. An initial population with *i* = 1,2,…,*k* = 4 unique sequence tags, indicated by green, purple, beige and blue color, respectively, undergoes a birth–death process (biological bottleneck; indicated by red arrows) for time *t* with division rate *β* [min^−1^] and death rate *δ* [min^−1^]. At the beginning of the experiment and at time *t*, *S_1_* cells are sampled, i.e. pass through a technical bottleneck (small opening in big grey bars). This could for example represent harvesting a set of cells during a time-lapse experiment. This changes the proportion of subpopulation *i* from *f_i_(t)* to *f_i,1_*. Following genome extraction (black loops), the genetic tag regions (colored triangles) are amplified by PCR, which we assume is unbiased. Hence, the proportion of the subpopulations do not change and remain *f_i,1_*. The amplified tag regions are then sequenced, which constitutes another technical bottleneck (grey bars), where *S_2_* sequence reads are sampled and the proportions are changed from *f_i,1_* to *f_i,2_.* (For interpretation of the references to color in this figure legend, the reader is referred to the web version of this article.)

Equations (6ab) prescribe how to estimate the division rate and death rate from CFU measurements and *N_B_* measurements assuming that the only contribution to the variance in the subpopulation proportions in equation (3) is due to a birth and death process. Another implicit assumption made with regard to equation (3) is that the variance is conditional on *f_i_(0)* which means that *f_i_(0)* is treated deterministically. However, a typical experiment involves additional technical bottlenecks that add to the variance in the subpopulation proportions. For example, sequencing is a technical bottleneck due to the limited capacity of the sequencing chip and sample preparation can impose bottlenecks. Additional technical bottlenecks include sampling the initial proportions which means that *f_i_(0)* in itself is a random variable. The aim of this section is to understand how to subtract the added variance in the subpopulation proportions due to technical bottlenecks so that the experimentally determined founder population size value is consistent with the assumptions made in deriving equations (6ab) for estimating the bacterial division rate and death rate. Equation (3) is central in this endeavor and whose derivation (see *2.3 RESTAMP - Bottleneck sensitivity of bacterial division rate and death rate estimates*) relied on the approximation < *f_i_|f_i_(0)*>≈ *f_i_(0)*, where < *f_i_|f_i_(0)* > is the mean tag proportions conditional on the tag distribution at *t* = 0. In this section *f_i_(0)* is treated as a random variable, hence < *f_i_*>=<*f_i_(0)* > by the law of total expectation [23]. Thus, for the derivation of equations (6ab) to be valid the initial proportions in equation (1) need to be substituted for the average initial proportion, <*f_i_(0)* > . Experimentally, we use triplicate samples of *f_i_(0)* to estimate the mean initial proportions of sequence tags, <*f_i_(0)*> (see *6.7-RESTAMP*). To separate the contributions from a birth–death process to *N_B_* and the contributions due to sampling bottlenecks we iteratively apply the law of total expectation and the law of total variance to propagate the variance in the frequency of sequence tags throughout the experiment illustrated in Fig. 2A (see *6.5* – *Correcting for technical bottlenecks by the iterative application of the law of total expectation and the law of total variance*). From this analysis we find that subtracting the added genetic variation due to *j =* 1,2,..,*m_I_* technical bottlenecks of size *I_j_* at time 0 and *j =* 1,2,..,*m_S_* technical bottlenecks of size *S_j_* at time *t*, can be achieved by analyzing the frequency of sequence reads according to

(7) $${<N}_{B}\left(t\right)>\approx \frac{1}{\frac{1}{k}\sum_{i=1}^{k}\frac{{\left(f_{i}\left(t\right)-{<f}_{i}\left(0\right)>\right)}^{2}}{{<f}_{i}\left(0\right)>\left(1-{<f}_{i}\left(0\right)>\right)}-\sum_{j=1}^{m_{S}}{<S}_{j}^{-1}>-\sum_{j=1}^{m_{I}}{<I}_{j}^{-1}>}$$

where <*S_j_*> is the j:th sequential mean sample size taken at time *t* and <*I_j_*> is the j:th sequential mean sample size at *t = 0* taken to estimate <*f_i_(0)*>. Equation (7) corresponds to the equation used in [8] to analyze data for *m_I_* = *m_S_* = 1 and is validated against simulations and experiments in section *3. Stochastic Simulations and Experimental Results*. The typical experimental setup for RESTAMP used in this work is illustrated in Fig. 2 which highlights the technical bottleneck events where *m_I_* = *m_S_* = 2.

### 2.3 RESTAMP – Bottleneck sensitivity of bacterial division and death rate estimates

Tight technical bottlenecks can affect growth and death rate determination despite corrections. How much technical bottlenecks affect rate determinations and the impact on experimental design remain unanswered questions. The variance in the subpopulation proportions determines the magnitude of the founder population size (equation (3)), and the total variance is approximatively the sum of the variances due to a birth–death process (*Var_BD_*) and a bottleneck event (*Var_B_*). Hence, if ${\mathrm{Var}}_{B}\left(f_{i}\right)/{\mathrm{Var}}_{\mathrm{BD}}\left(f_{i}\left(t\right)\right)$ approaches ∞ then ${\mathrm{Var}}_{\mathrm{BD}}\left(f_{i}\left(t\right)\right)$ ≪ ${\mathrm{Var}}_{B}\left(f_{i}\right)$ and the technical bottleneck event becomes dominating. As a numerical and artificial example, if *Var_B_(f_i_)* is 100 and *Var_BD_(f_i_)* is 1 then the total variance is 101. Suppose that the error in estimating the sample sizes in equation (7) has an error rate of 10% due to experimental noise. Thus, one might conceivably estimate the bottleneck correction term to be 90. The variance due to the birth–death process is then estimated as (1 + 100)-90 = 11. This overestimates the variance by one order of magnitude and results in a one order of magnitude underestimation of the founder population size values and approximately a one order of magnitude overestimate in the rates according to equations (6ab). In another case, one might estimate the correction term to be 110 which would lead to a negative founder population size value. While negative founder population size values cannot be used to estimate rates, they are a strong indicator of the presence of very stringent technical bottlenecks. Considering the reverse situation where *Var_BD_(f_i_)* is 100 and *Var_B_(f_i_)* is 1, the impact of experimental noise due to technical bottlenecks is negligible. Thus, we define a bottleneck sensitivity measure, *s_B_(t)* = ${\mathrm{Var}}_{B}\left(f_{i}\right)/{\mathrm{Var}}_{\mathrm{BD}}\left(f_{i}\left(t\right)\right)$.

The variance in the subpopulation proportions due to a birth–death process is given by equation (4) and is derived in *5.4 - The variance in the proportion of cells with respect to repetitions for a birth*–*death process*. For a technical bottleneck event, modeled as a multinomial random sampling process, the variance is ${\mathrm{Var}}_{B}\left(f_{i}\right)=f_{i}\left(0\right)\left(1-f_{i}\left(0\right)\right)/N_{B}$ where the sample size is *N_B_*. The bottleneck sensitivity measure is therefore given by

(8) $$s_{B}\left(t\right)=\frac{{\mathrm{Var}}_{B}\left(f_{i}\right)}{{\mathrm{Var}}_{\mathrm{BD}}\left(f_{i}\left(t\right)\right)}\approx \frac{r}{\left(\beta +\delta \right)\left(1-e^{-rt}\right)}\frac{N(0)}{N_{B}}$$

Equation (8) shows that the bottleneck sensitivity, *s_B_(t)*, increases with smaller sample sizes (*N_B_*) as expected. What might be less intuitive is the dependence of *s_B_(t)* on the total population size at *t* = 0. This results from the variance in the proportion of cells, due to a birth–death process, being smaller for larger population sizes (equation (4)). Importantly, the total population size at *t* = 0, *N(0)*, is implicitly expressed as per unit volume, which means that *s_B_(t)* is also a quantity that depends on the volume. Since CFUs are typically reported as per ml, we define *s_B_(t)* to be in a volume of 1 ml meaning that the value for *N(0)* to be put into equation (8) is the CFU count per ml. We perform control experiments to find a threshold value for *s_B_(t)* below, which we expect to result in accurate rate estimates in section 3. Another feature of equation (8) is that sampling bottlenecks can become much more important to account for than the sequencing bottleneck. For example, consider a sampling bottleneck where 2x10^5^ cells are sampled for determining the founder population size. After genome extraction, shearing, and PCR amplification, the sequence tags are sequenced on a chip with a capacity of the order of 2x10^7^ sequence reads. Hence, the variance in the subpopulation proportions due to the sampling bottleneck are 100 times higher than for the sequencing bottleneck. Failure to correct for the sampling bottleneck as prescribed by equation (7) will result in underestimating the founder population size due to a birth–death process and consequently overestimating the division rate and death rate (equations (6ab)).

Equation (8) also shows that technical bottlenecks become more dominating for shorter times, or for stationary-like cells where the division rate and death rate are small in magnitude. Shorter times and smaller rates will therefore magnify technical bottleneck effects and could potentially lead to overestimating the rates. Importantly, the bottleneck sensitivity measure depends on experimentally controllable parameters. Using an estimate of the individual rates as *β*≈*r*, *δ*≈0 for a growing population of cells or *β*≈*0*, *δ*≈*r* for a dying population of cells we can plan the experiment so that the sensitivity is minimized and the accuracy of rate estimates maximized.

## 3 Results – stochastic simulations and experiments

**Fig. 3:**
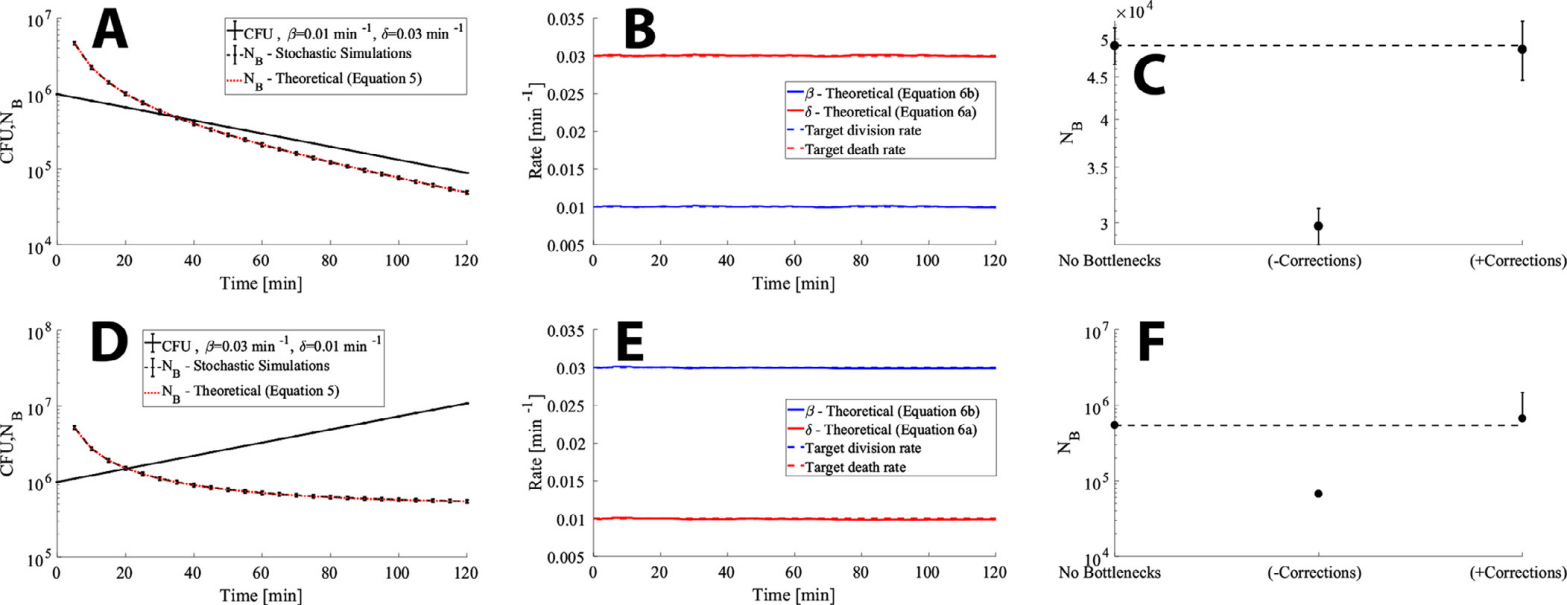
The theoretical framework for RESTAMP agrees well with stochastic tau-leaping simulations. (A) A population of *k* = 1000 distinguishable cells undergo a random birth–death process with *δ* = 0.03 min^−1^ and *β* = 0.01 min^−1^ for 120 min with a geometrical tag distribution at *t* = 0 with the probability parameter set to 1/1000. The stochastic tau-leaping simulations were ran using StochKit2 [24] with a time-step 0.01 for 100 iterations. A custom script for generating the input file to StochKit2 is available on SourceForge (see *6 – Code*). The proportion of subpopulations was determined for each time point and the founder population size was calculated using equation (1). The mean founder population size and the standard deviation were next determined and plotted as a function of time (black dashed line). The CFU were calculated by summing the *i* = 1,2,…,*k* subpopulations at each time point after which the mean (over iterations) CFU and the standard deviation were determined (black solid line). The theoretical founder population size values were calculated using equation (5) (red dotted line). (B) Using the mean CFU and mean NB values illustrated in (A) we estimate the division rate and death rate over time using equations (6ab). (C) The population of cells at *t* = 120 min undergo two sequential multinomial random sampling events (technical bottlenecks) where the sample sizes *S_1_* = 10^5^ and *S_2_* = 10^6^ were taken. The inoculum (population at *t* = 0) also underwent two sequential random sampling events where the sample sizes *I_1_* = *I_2_* = 10^6^ were taken. The founder population size was then calculated according to equation (1) which does not include bottleneck corrections and equation (7) which includes bottleneck corrections. The target founder population size without any technical bottlenecks is also shown and corresponds to the founder population size at *t* = 120 min before sampling. (D-F) Same as (A-C) except *δ* = 0.01 min^−1^ and *β* = 0.03 min^−1^. The lower bound for the standard deviation in (F) is not shown on a log scale since it is negative, i.e. the standard deviation is larger than the mean *N_B_* value. The mean and the standard deviation of the founder population size values are <*N_B_*>={5.5 × 10^5^, 6.6 × 10^4^, 6.1 × 10^5^ and Std(*N_B_*)={2.5 × 10^4^, 3.2 × 10^3^, 7.1 × 10^5^} for the results plotted in (F). The corresponding values for (C) are <*N_B_*>={4.9 × 10^4^, 3.0 × 10^4^, 4.9 × 10^4^} and Std(*N_B_*)={2.5 × 10^3^, 1.5 × 10^3^, 4.1 × 10^3^}. All scripts for reproducing these results are provided on SourceForge (see *6 – Code*). (For interpretation of the references to color in this figure legend, the reader is referred to the web version of this article.)

We first test the theory developed in section 2 by comparing against stochastic tau-leaping simulations [24] for the case of cells dying on average with *δ* = 0.03 min^−1^, *β* = 0.01 min^−1^ (Fig. 3A, 3B and 3C) and for cells growing on average with *δ* = 0.01 min^−1^, *β* = 0.03 min^−1^ (Fig. 3D, 3E and 3F) for *t =* 120 min. The sequence tag distribution at *t* = 0 is geometric with the probability parameter set to 1/1000 for *k* = 1000 unique sequence tags, which results in an inoculum size, *N(0)*, of approximately 10^6^ cells. This is sufficient information to calculate the theoretical average founder population size (equation (5)) as a function of time, shown as red dotted lines in Fig. 3A and 3D. The corresponding stochastic tau-leaping simulations were ran for 100 iterations with the founder population size calculated using equation (1) for each iteration. The mean and the standard deviation for both the *N_B_* values (black dashed line) and the CFU values (black solid line) where subsequently calculated and plotted in Fig. 3A and 3D. Fig. 3A and 3D show an excellent agreement between the theoretical average founder-population size and the simulated average. Next, we use equations (6ab) to determine the division and death rate for both cases (Fig. 3B and 3E). The estimated death rate (solid red line) and division rate (solid blue line) agree very well with the target rates (dashed lines), with the deviations being negligibly small.

Lastly, we tested equation (7) which prescribes how to correct for technical bottlenecks, including sampling from a larger volume and sequencing with a limited chip capacity. The population at *t* = 120 min and the inoculum, the population at *t* = 0, undergoes two random sampling events (*m* = 2) where *I_1_* = *I_2_* = *S_2_* = 10^6^ and *S_1_* = 10^5^. The ideal *N_B_* value without any added technical bottlenecks, the estimated *N_B_* value without correction terms (equation (1)) and the estimated *N_B_* value with correction terms (equation (7)) are plotted in Fig. 3C and 3D. The results show excellent agreement between the theoretical mean *N_B_* value and the *N_B_* value as calculated using equation (7) with technical bottleneck corrections. However, we observe that the standard deviation relative to the ideal case without technical bottlenecks increase more when a sample is very small relative to the total population size. Notably, the standard deviation can even be larger than the mean founder population size value (Fig. 3F). It is therefore important to carefully design experiments aimed at estimating division rates and death rates to minimize this source of error. Figure Supplementary Fig. 2 shows additional stochastic simulation results where the accuracy and precision in rate estimates are compared to the corresponding bottleneck sensitivities. The results show that the accuracy and precision for RESTAMP rate estimates increase as the magnitude of the bottleneck sensitivity decrease.

Next, we tested the RESTAMP method by devising a control experiment where we controled death rates (see *5.1 - Emulating a death process by sampling*). We aimed to emulate a pure death process by sampling a volume *Δv_t_* = *Δv_0_e^-δt^* from a flask that contained *E. coli* MG1655 cells tagged with *k* = 1000 unique, 30 bp long sequence-tags that are fitness neutral as experimentally verified (Figure Supplementary Fig. 3). The target division rate is 0 and the death rate and time points can be freely chosen. We set a high target death rate of *δ* = 0.1 min^−1^ and a low target death rate of *δ* = 0.015 min^−1^. The time points were set to *t*={20,25,30,35,40} min. The sampled volume of the inoculum at *t* = 0, *Δv_0_*, was 1 ml and equation (7) was used to calculate the founder population size values for each time point with *m_I_ = m_S_* = 1 bottleneck corrections due to the limited sequence chip capacity. The specific instantiation of equation (7) used to analyze the sequence reads for this experiment is therefore

(9) $$N_{B}\left(t\right)\approx \frac{1}{\frac{1}{k}\sum_{i=1}^{k}\frac{{\left(f_{i}\left(t\right)-{<f}_{i}\left(0\right)>\right)}^{2}}{{<f}_{i}\left(0\right)>\left(1-{<f}_{i}\left(0\right)>\right)}-<S_{1}^{-1}>-<I_{1}^{-1}>}$$

**Fig. 4:**
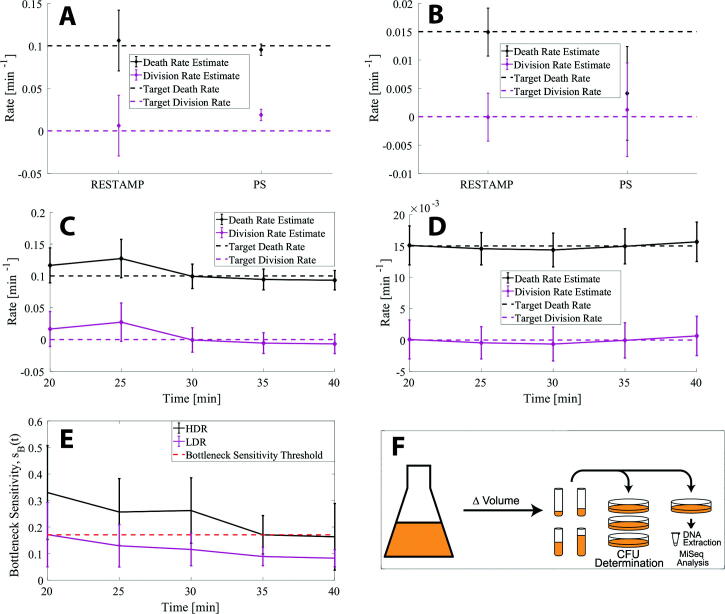
The death rates and division rates in an emulated death process are accurately determined by the RESTAMP and PS methods. A pure death process is emulated by sampling different volumes from a starting culture to correspond to different time points in a death process (see *5.1 – Emulating a death process by sampling*). (A) The target division rate for a pure death process is 0 (magenta dashed line) and the target death rate was set to 0.1 min^−1^ (black dashed line). The time points for the RESTAMP experiment were set to *t* = {20,25,30,35,40} min and 3 repetitions of the experiment were performed. The diamond marker shows the mean estimated rate in a sample size of 15 rates determined for each time point and experiment. The bars show the standard error of the mean. The chosen time points for the plasmid segregation experiment were set to *t* = {20,40,60,80} min and 3 repetitions of the experiment were performed. (B) Same as (A) except the target death rate was set to 0.015 min^−1^ and the time points for the plasmid segregation experiments were set to *t*={20,25,30,35} min. (C) Time resolved division rate and death rate estimates for the high death rate case where the target death rate is 0.1 min^−1^ (black dashed line) and the target division rate is 0 (magenta dashed line). For each time point, the mean value (diamond marker) and the standard error of the mean are shown. (D) Time resolved division rate and death rate estimates for the high death rate experiment, where the target death rate is 0.015 min^−1^ (black dashed line) and the target division rate is 0 (magenta dashed line). For each time point, the mean value (diamond marker) and the standard error of the mean are shown. (E) The bottleneck sensitivity was calculated using equation (8) for the individual high death rate (HDR *–* black solid line) and the low death rate experiments (LDR *–* magenta solid line) (Figure Supplementary Fig. 4) where the plot shows the mean bottleneck sensitivity ± S.D. A threshold value of *s_B_(t)* = 0.17 for the bottleneck sensitivity was set to correspond to robust rate estimates (red dashed line) at *t* = 35 min. (F) A simple schematic of the experiment. Different size volumes (*Δv_t_*) are sampled from an Erlenmeyer flask which contains either a population of cells with a sequence tag (STAMP) or a population of cells with an identifiable plasmid (PS) in LB media. The size of the volumes was determined so as to emulate a pure death process (see *5.1 – Emulating a death process by sampling*). Colony forming units (CFUs) were determined by serial dilution for the largest volume (corresponding to *t* = 0) and extrapolated to the pre-determined time points in (C-D). For the RESTAMP method, the genomes were extracted and tag frequencies were determined by next-generation sequencing. For the PS method the fraction of cells carrying the conditionally replicative plasmid were determined by selective plating. The experiments were repeated biologically independently three times. Rate estimates for all trials at each time point and experimental CFU and *N_B_* values are available for download on SourceForge (see *6 – Code*). (For interpretation of the references to color in this figure legend, the reader is referred to the web version of this article.)

In addition, matching a death-process with a multinomial random sampling process requires scaling the *N_B_* values by the factor 1/(1-*p*) where *p = e^-δt^* (see *5.1 - Emulating a death process by sampling*). The mean founder population size at each time point and the corresponding mean CFU were substituted into equations (6ab) to estimate the bacterial division rate and death rate. The mean CFU at *t =* 0 was determined by serial dilution. The CFU was determined as < *N(0)* > *e^-δt^* at time *t*. We also performed the same experiment using plasmid (*pAM34-Plac*) containing cells and the established plasmid segregation method to estimate rates (*Materials and Methods – Plasmid Segregation*) [12]. Fig. 4F illustrates a simple schematic of the experimental workflow.

Fig. 4A shows the mean estimated rates and the standard error of the mean, at all time points, for the high death rate experiment. The corresponding data for the low death rate experiment is shown (Fig. 4B). The mean rate estimates (diamond markers) agree well with the target death rates and division rate of 0 for both methods. However, RESTAMP does have a slight propensity to overestimate the rates, particularly for the high death rate case. From the time-resolved RESTAMP rate estimates we see that much of the contribution to the noise is for the shorter time points (Fig. 4C), where we also observe larger fluctuations in the mean rate estimates. In contrast, the time-resolved RESTAMP rate estimates for the low death rate experiment are robust (Fig. 4D). We exploit this difference between the high death rate and the low death rate experiments to define a threshold for the sensitivity measure *s_B_(t)* (equation (8)), below which we expect rate estimates to be accurate and robust against making an error in estimating the technical bottleneck correction terms in equation (7). The sensitivity measure is plotted in Fig. 4E for the high death rate (black line) and the low death rate (magenta line) experiments, where the parameter *N_B_* was set to 2x10^7^ (i.e. the sequencing bottleneck) in equation (8). The total population size at *t = 0, N(0),* were experimentally determined to be 5.8x10^5^ CFU/ml for the high death rate experiment and 7.7x10^4^ CFU/ml for the low death rate experiment. The graph shows that *s_B_(t)* is larger for the high death rate experiment, primarily a consequence of *N(0)* being larger by a factor 7.5. A larger *N(0)* means that the variance in the subpopulation proportions due to the death process is smaller. Hence, the variance due to technical bottlenecks becomes more dominating and the sensitivity in the rate estimates increases. The death rate estimates for the high death rate case are robust after 25 min, however we do notice large fluctuations for the bottleneck sensitivity value at *t* = 25 min and at *t* = 30 min. . Therefore, we set a threshold *s_B_(t)* = 0.17, corresponding to the bottleneck sensitivity value at *t* = 35 min. From the definition of *s_B_(t)* in equation (8), this means that the variance in the frequency of subpopulation *i* due to random birth–death processes in a volume of 1 ml should be at least 5 times larger than the variance due to sampling. Fig. S4 shows the rate estimates and the bottleneck sensitivities for the individual replicates at all time points.

**Fig. 5:**
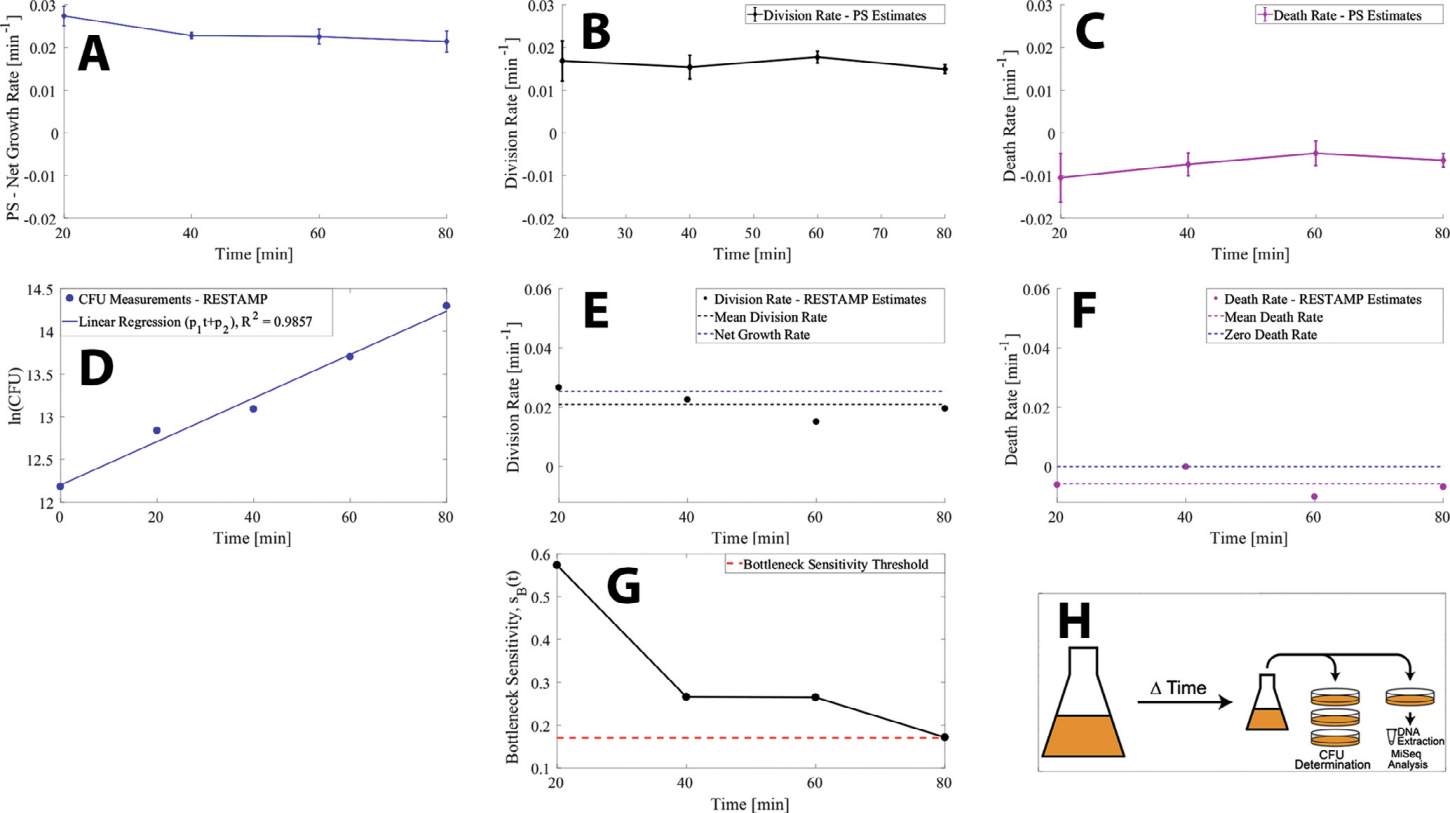
Estimated division rates and death rates for RESTAMP and PS for cells growing in LB media. (A) Net growth rate estimates at each time point for three repetitions of a plasmid segregation experiment. The diamonds and bars show the standard error of the mean. (B) Estimates of average division rates using the plasmid segregation method (diamonds) where the bars show the standard error of the mean. (C) Estimates of average death rates using the plasmid segregation method (diamonds) where the bars show the standard error of the mean. (D) A linear regression of the natural logarithm of the CFUs versus time. The slope *p_1_* is the average net growth rate with the 95% confidence interval given in parenthesis where *p_1_* = 0.02547 (0.01984, 0.03111) min^−1^ and *p_2_* = 12.2 (11.93, 12.48) min^−1^. (E) Estimated division rates using the RESTAMP method at each time point of the experiment (black circles). The black dashed line is the average division rate and the blue dashed line is the net growth rate. (F) Estimated death rates using the RESTAMP method (magenta circles). The dashed magenta line is the average estimated death rate and the blue line is a death rate of 0. (G) The bottleneck sensitivity (y-axis, black solid line)) for sequencing was calculated according to equation (8) where *r = β =* 0.025 min^−1^, *t*={20, 40, 60, 80} min, *N(0)* = 1.95 × 10^5^ CFU/ml and *N_B_* is the mean sample size on the sequencing chip (*S_2_*) where <*S_2_>* = {8.643 × 10^5^, 1.16 × 10^6^, 9.4751 × 10^5^, 1.3145 × 10^6^}. The red dashed line corresponds to the bottleneck sensitivity threshold, 0.17. (H) A simple schematic of the experiment also illustrated in Fig. 1C. Samples were taken from an Erlenmeyer flask, which contains either a population of cells with a sequence tag (STAMP) or a population of cells with an identifiable plasmid (PS) in LB media after growing for time *t*. Colony forming units (CFUs) were determined by serial dilution for all samples in triplicates. For the RESTAMP method, the genomes were extracted and tag frequencies were determined by next-generation sequencing. For the PS method the fraction of cells carrying the conditionally replicative plasmid were determined by selective plating. The experiments were repeated biologically independently three times. Rate estimates for all trials at each time point and experimental CFU and *N_B_* values are available for download on SourceForge (see 6 *– Code*). (For interpretation of the references to color in this figure legend, the reader is referred to the web version of this article.)

Next we perform an experiment for bacteria growing in complex media (LB) where we do not control the division and death rates. Fig. 5H illustrates a simple schematic of the experimental workflow. Fig. 5A shows the mean net growth rate and the standard error of the mean of three repetitions of the plasmid segregation experiment where the bacteria grow for a time *t*={20,40,60,80} min. The mean net growth rate is stable at approximately 0.025 min^−1^ corresponding to a generation time of 28 min. Fig. 5B and 5C show the plasmid segregation division and death rate estimates, respectively. Here we see that the division rate is close to the net growth rate, meaning that the bacteria are not dying. The death rates are close to 0, although may be estimated as negative due to experimental noise. Hence, we expect that the RESTAMP rate estimates produce the same result with no death and a division rate close to the net growth rate. Fig. 5D shows the natural logarithm of the CFUs as estimated in the RESTAMP experiment at the time points *t*={20, 40, 60, 80} min. The slope of the regression line is an estimate of the net growth rate of 0.025 min^−1^. Fig. 5E and 5F show the division and death rate estimates for each time point, respectively. Here we see that the division rate estimates are accurate where the mean division rate over all time points (black dashed line) correlate very close to the net growth rate (blue dashed line). Likewise, we see that the death rate estimates are close to 0, and can potentially be estimated as negative due to experimental noise as discussed in section *2.3 RESTAMP - Bottleneck sensitivity of bacterial division rate and death rate estimates*. Fig. 5G shows the magnitude of the experimental bottleneck sensitivities for sequencing where the dashed red line correspond to the bottleneck sensitivity threshold, *s_B_(t)* = 0.17. Since the bottleneck sensitivities exceed the threshold value we expect that the rate estimates are more sensitive to making errors in estimating the bottleneck correction terms in equation (7). We confirm this by reanalyzing the data without bottleneck correction terms using equation (1) and comparing with the rate estimates with bottleneck correction terms (Figure Supplementary Fig. 5). Important to note is that the RESTAMP rate estimates need to be closely integrated with the experimental protocol. In this experiment we have two technical bottleneck events (Fig. 2), the first from sampling 200 μl and the second from sequencing (Fig. 5H). Therefore, *m_I_ = m_S_* = 2 and the specific instantiation of the equation used to calculate *N_B_* values (equation (7)) is

(10) $$N_{B}\left(t\right)\approx \frac{1}{\frac{1}{k}\sum_{i=1}^{k}\frac{{\left(f_{i}\left(t\right)-{<f}_{i}\left(0\right)>\right)}^{2}}{{<f}_{i}\left(0\right)>\left(1-{<f}_{i}\left(0\right)>\right)}-<S_{1}^{-1}>-<I_{1}^{-1}>-<S_{2}^{-1}>-<I_{2}^{-1}>}$$

where <*I_1_>* = 3.9x10^4^ CFUs and <*S_1_>* = {7.5 × 10^4^, 9.7 × 10^4^, 1.8 × 10^5^, 3.2 × 10^5^} CFUs corresponding to each time point *t*={20,40,60,80} min for the sampling bottleneck. For the sequencing bottleneck we have <*I_2_>* = 1.04 x10^6^ sequence reads and <*S_2_>* = {8.643 × 10^5^, 1.16 × 10^6^, 9.4751 × 10^5^, 1.3145 × 10^6^} sequence reads.

## 4 Discussion

Powerful methods have been devised to investigate the detailed population dynamics of pathogens in order to gain insight into the disease causing mechanisms and establish guiding principles and strategies for disease prevention. Typically, these methods are based on tracking and identifying a marker that loses signal strength as the cells divide or die. Plasmid segregation (PS) have been shown to be a very capable method which uses conditionally non-replicative plasmids as the marker [12]. Since the proportion of cells that contain a plasmid decrease exponentially with time (*Materials and Methods – Plasmid Segregation*), the PS method and other marker-based techniques work best during a short time window. It can also be experimentally challenging to ensure that the accessory genes or fluorescent markers of the plasmids do not change the wild-type division and death rates, especially in the context of within-host infection models. Methods such as WITS can overcome some of the limitations of the plasmid segregation method [1]. The WITS method infer migration rates, division rates and death rates based on changes in the composition of tags in the total population. The tradeoff is that WITS is more sensitive to technical bottlenecks, e.g. sampling and sequencing, as these influence the variation in the genetic composition of the total population [17]. However, it should be noted that previous work have taken steps towards detecting technical bottlenecks in [25], [26] which is summarized in [27]. In addition, the mathematical analysis of WITS data relies on the assumption that the tags are initially evenly distributed [1], [2], [3]. Consequently, these studies are typically constrained to using ~ 10 tags, which limits the accuracy in the rate estimates.

In this work, we develop the RESTAMP method (Rate Estimates by Sequence Tag Analysis of Microbial Populations) that takes into account, and corrects for, the impact of technical bottlenecks. The mathematical framework for RESTAMP is constructed on top of the experimental STAMP method [8] and provides a simple way to aggregate information about many sequence tags into a single measure; i.e. the founder population size. Hence, our method can handle any number of DNA sequence tags, limited only by the sequence chip capacity. Furthermore, we show that the average founder-population size is independent of the initial tag distribution. This simplifies the process of estimating rates for the experimentalist, as the sample does not require an exact composition of tags.

The independence of the mean founder population size on the sequence tag distribution at *t* = 0 (equation (5)) relies on a fixed initial tag distribution, *f_i_(0)*. Ideally, this means that the samples taken to determine mean founder population size values exactly reflect the sequence tag distribution of the population they were sampled from, regardless of the initial distribution. However, it is experimentally challenging to completely remove the influence of sampling on tag distributions. Thus, to minimize the effect of sampling on the accuracy of the rate estimates, multiple samples are taken from the same culture. Ultimately, we find that the impact of this variation is minimal since we get reasonable rate estimates as validated by control experiments and by comparison with the PS method (Fig. 4, Fig. 5).

The successful application of the bottleneck corrections is contingent on an accurate measurement of the CFUs used to determine the sample sizes. Severely underestimating the sample size can result in calculating a negative founder population size value. Even with an accurate CFU estimate, there is also a chance to produce negative founder-population size values in the presence of stringent technical bottlenecks. This is due to the noise from random division and death events being overwhelmed by the magnitude of stochastic variation induced by technical bottlenecks (see *2.3 RESTAMP – Bottleneck sensitivity of bacterial division rate and death rate estimates*). We used this to define a bottleneck sensitivity measure, *s_B_(t)*, in the rate estimates as the ratio between the variance in the subpopulation proportions due to a birth–death process and the variance due to a multinomial random sampling event used to model a technical bottleneck. By devising a control experiment, we *a priori* set the target death rate by emulating a death processing by sampling (see *5.1 – Emulating a death process by sampling*) and find a threshold for the sensitivity measure, below which we expect the rate estimates to be robust and accurate (Fig. 4). For the experimental results illustrated in Fig. 5 we find that the bottleneck sensitivity is larger than the sensitivity threshold. We therefore expect that the rate estimates are more sensitive to making an error in the bottleneck correction terms in equation (7). We confirm this by comparing the rate estimates with and without bottleneck correction terms in Figure Supplementary Fig. 5.

**Supplementary Fig. 1:**
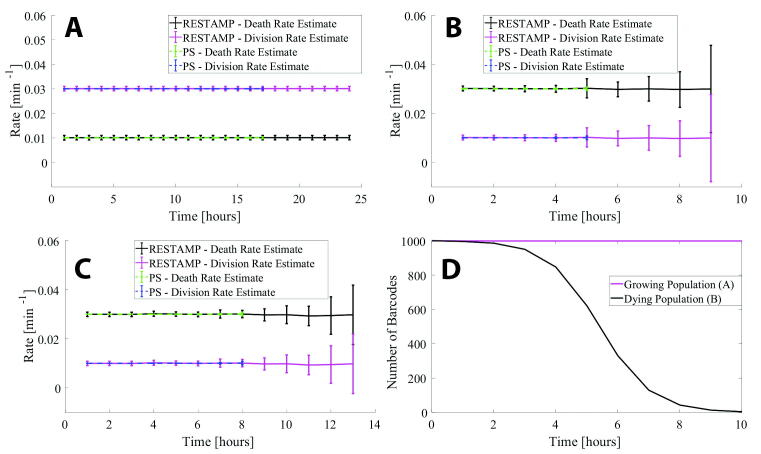


**Supplementary Fig. 2:**
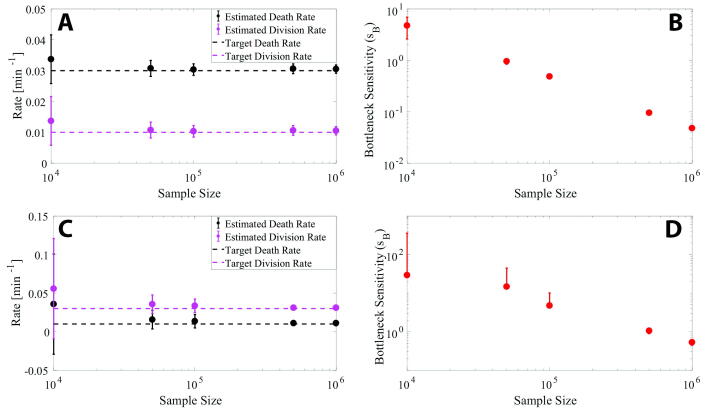


**Supplementary Fig. 3:**
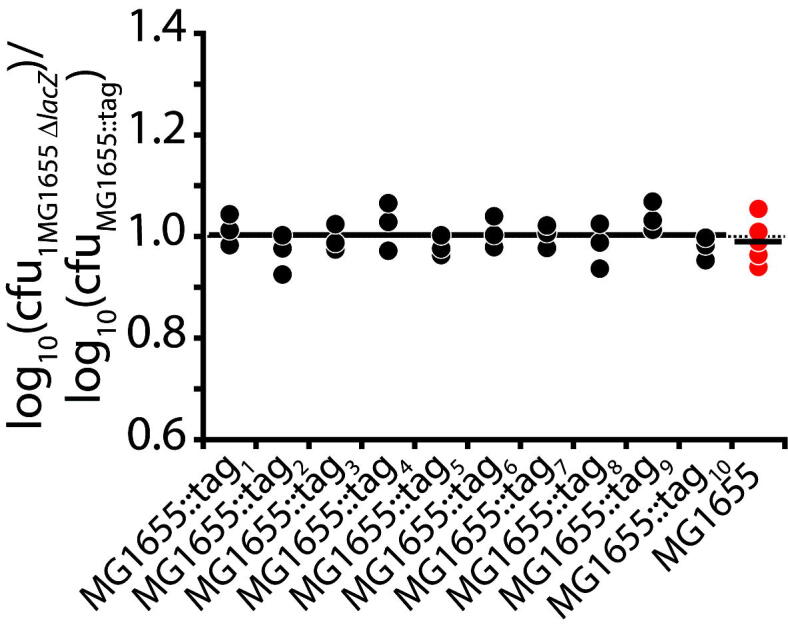


**Supplementary Fig. 4:**
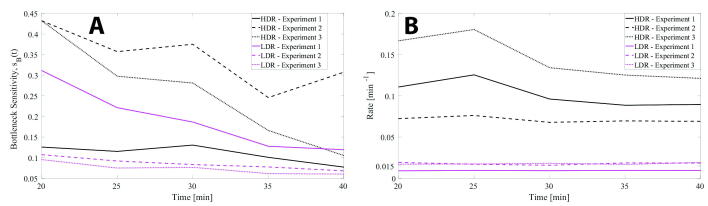


**Supplementary Fig. 5:**
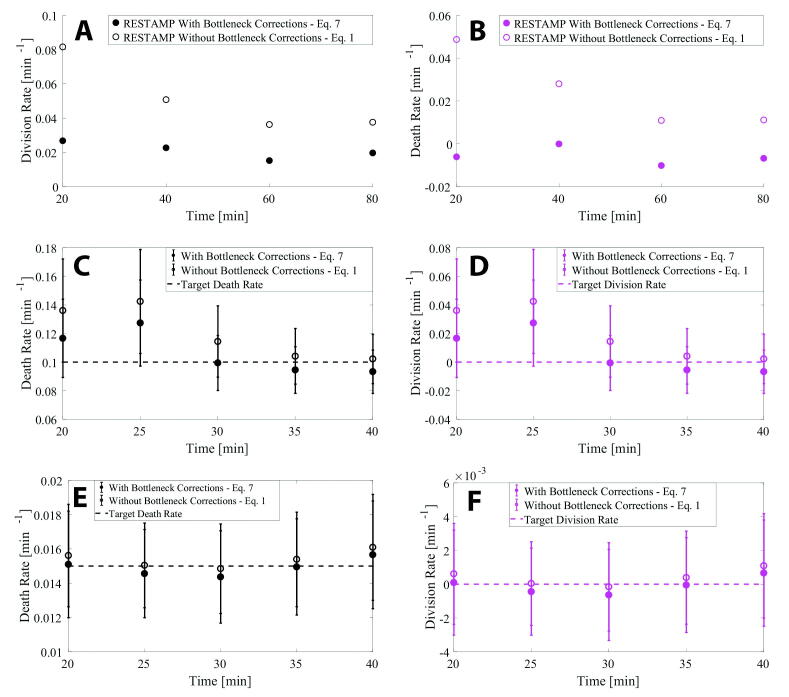


The sensitivity measure predicts an increasingly accurate rate estimate for longer observation times. In control experiments, we find that the relative fluctuations (the standard error of the mean relative to the mean) are 16–23% for the high death rate case (*δ* = 0.1 min^−1^) and 18–21% for the low death rate case (*δ* = 0.015 min^−1^) (Fig. 4). We find that the magnitude of the relative fluctuations are more stable at 18–21% at each time point as expected based on the shallow slope of the bottleneck sensitivity measure versus time (Fig. 4E - magenta line). By comparison with the PS method, RESTAMP mean rate estimates are slightly closer to the target division and death rates. In terms of the uncertainty in the rate estimates, RESTAMP performs equally well as the plasmid segregation method. However, the advantages of RESTAMP comes into the forefront when it is experimentally challenging to assess whether the plasmids are fitness-neutral or where long observation times (Figure Supplementary Fig. 1) are needed; e.g. in studying within-host population dynamics.

In Figure Supplementary Fig. 1 we investigate the precision and accuracy of rate estimates as a function of time up until 24 h for both RESTAMP and PS by performing stochastic tau-leaping simulations. If the cells are growing, on average (Figure Supplementary Fig. 1A) we observe a practically unlimited observation time with RESTAMP while PS is limited to ~ 17 h. The reason is that the plasmid containing cells are unaffected by division events and are lost on average at a rate proportional to $e^{-\delta t}$. However, the precision and the accuracy of the rate estimates using the PS method are very robust during the time window where PS works. This remains true for the PS method also in the case when cells are dying (Figure Supplementary Fig. 1B), where on average the observation time is reduced to ~ 5 h as the death rate for the cells is increased. The RESTAMP observation time also becomes limiting for this case where we observe a deterioration in the precision of the rate estimates beginning at ~ 5–6 h, a consequence of lost sequence tags. To calculate the fraction of unique sequence tags that survives until time *t* in a birth–death process, we adopted the mathematical framework for a transposon insertion sequencing experiment [21]. The variables are reinterpreted in the context of RESTAMP where the number of transposon insertion sites correspond to the number of barcodes (*k* = 1000). The fitness coefficients (*w_i_*) are set to 1 and the equation for the extinction probability for a birth–death process was used (equation (6) in [21]). The extinction probabilities were subsequently used to calculate the mean reduction in library complexity (equation (8) in [21]), interpreted as the fraction of unique sequence tags that survives until time *t*. Upon multiplying the fraction of sequence tags by *k* we get the number of unique sequence that survives until time *t*. This is plotted in Figure Supplementary Fig. 1D for both the case of cells growing on average and for cells dying on average. For the former case we do not observe any loss of tags while for the latter the number of tags decrease as a function of time where there is less than 400 tags at 5–6 h. In principle, one could increase the initial population size to drive the observation times to be longer for both methods. For example, in Figure Supplementary Fig. 1C the inoculum size was increased by a factor of 100 to *N(0)* = 10^8^ for the case of cells dying on average.

In summary, it might be advantageous to increase the number of unique sequence tags prior to executing a RESTAMP experiment to buffer against loss of accuracy depending on whether severe death events are expected. To aid in this decision, this type of analysis could be used to estimate the expected number of extinct sequence tags, e.g. in within-host infection models, from the CFU time-course data by setting *r*≈*δ* for cells dying on average.

The RESTAMP method abstracts away the details of the underlying stochastic dynamics that drive birth–death processes and simplifies the analysis of an arbitrary number of sequence tags by providing an explicit equation relating the division and death rates with the average *N_B_* and CFU values (equations (6ab)). It was previously shown that the accuracy of *N_B_* estimation can be improved by increasing the number of sequence tags [8]. Hence, there is potential for the accuracy of the rate estimates to improve with respect to the founder population size. However, there is a limit to this improvement due to other unavoidable sources of experimental errors. For example, the PCR amplification step might not be unbiased and error in CFU determination is heavily influenced by the experimentalist’s consistency in methodology (e.g. sample dilution, time to plate samples, counting). We minimized potential technical bottlenecks due to PCR by minimizing the number of amplification cycles and performing the amplification in triplicate and pooling the results. Our experimental results suggest that the best way to increase the accuracy is to take the average of multiple rate estimates as the average rates accurately approximate the target rates (Fig. 4AB).

The quantitative analysis of WITS data typically relies on expressing the dynamics in the form of a master equation, i.e. an equation for the probability of having a certain number of cells at a particular time point, whereby the rates are inferred using maximum likelihood estimates [1], [2], [3]. In contrast, RESTAMP abstracts away the details with respect to the master equation that drives the stochastic population dynamics. It is centered on analyzing the frequency of sequence tags by defining a particular function of the frequencies, e.g. equation (1) for the founder population size, such that it becomes relatively simple to tie it to experiments and correct for unavoidable technical bottlenecks.

A recent alternative approach employed the moment-closure method where a system of ordinary differential equations for the mean, variance, and covariance in the subpopulation sizes are solved for, and the rates inferred by adopting an appropriate divergence measure between the WITS data and the generated moments [7]. Likewise, the RESTAMP approach for inferring rates also depends on higher order moments, namely the variance in the proportion of subpopulations (equation (3)). Theoretically, the calculation of the average founder population size can be integrated with the moment-closure framework which would allow RESTAMP to also estimate migration rates in addition to division and death rates. By expressing the mean founder population size in terms of the first and second moments, we can expand to a multi-compartment model with arbitrary topology and location dependent division, death, and migration rates. This approach would allow RESTAMP to determine rates for bacteria that are not strictly growing exponentially, e.g. logistic growth, in multiple compartments and is best studied in the context of a within-host infection model.

Nevertheless, the RESTAMP framework as is can be used to provide a low-resolution picture of in vivo dynamics by treating the animal model as a single compartment. The first step in using RESTAMP to plan an in vivo or an in vitro experiment is to measure CFUs over time, i.e. a growth curve. The growth conditions for this experiment must be the same as the growth conditions that will later be used to determine division and death rates. It is not necessarily required to use the barcoded library of cells, as long as the used strain and the final tagged library have the same division and death rates, e.g. when the used tags are fitness neutral and the CFUs over times are measured with the untagged parental strain. Our model is restricted to exponential growth or decay. The CFU over time data can be used to check if the growth conditions fulfill this requirement and given a certain inoculum size, the time interval of exponential growth or decay can be determined. In this interval, the net growth rate *r* can be estimated. Next, it is necessary to identify whether the environment is particularly hostile to the cells. If there is a sharp decrease in the CFUs over time, then it might be necessary to increase the number of identifiable subpopulations (*k*) to buffer against barcode extinction events. The number of identifiable subpopulations should be at minimum *k =* 400–500 [8]. Whether this is a necessity can be checked by setting *r*≈*δ* and using the rationale as discussed above to plot a graph corresponding to Figure Supplementary Fig. 1D and read out the number of tagged subpopulations that survives until time *t*. The next step is to identify the number of technical bottlenecks in the experiment that all samples are subjected to, i.e. at time 0 (*m_I_*) and at time *t* (*m_s_*) (Fig. 2). In principle, all technical bottlenecks can be accounted for. However, in this work we only consider the two (*m_s_* = *m_I_* = 2) major technical bottlenecks, which typically are the harvesting of the cells from the growth environment and the limited sequencing depth of next-generation sequencing. This will normally remain true in an in vivo experiment, where all the cells are harvested from the organ of interest and then sampled and loaded onto a sequencing chip. The next step is to estimate the sample sizes for each technical bottleneck, e.g. the number of harvested cells (*S*_1_, *I*_1_) or the number of generated sequences (*S*_2_, *I*_2_). These are required to calculate the bottleneck sensitivity, *s_B_(t)*. Within the constraints of the experimental setup, these can be freely chosen. At this point the experimenter only needs an estimate of the division rate *β* and the death rate *δ* to calculate *s_B_(t)* for each sample by using equation (8). The number of cells per ml at the beginning of the experiment is known (*N(0)*), the net growth rate is known (*r*), the sampling time is known (*t*) and the sample size is known (*N_B_*). For a first estimate the (*β + δ*) term in equation (8), one can set *r*≈*δ* for a dying population of cells or *r*≈*β* for a growing population of cells. A better approach would be to calculate *s_B_(t)* over a range of biologically plausible division and death rates. For example, if the minimum expected time until the population doubles in size is ~ 15 min then the maximum division rate is ln(2)/15 min^−1^ ~ 0.046 min^−1^. Likewise, if the minimum expected time until the population halves in size is 5 min then the maximum death rate is ln(2)/5 min^−1^ ~ 0.14 min^−1^. If this calculation shows an *s_B_(t)* value that exceeds the threshold of *s_B_(t)* = 0.17 (see justification above, Fig. 4E) then the experimenter can change the experimental setup, i.e. lower the initial concentration (*N(0)* per ml), sample more cells (increase *N_B_*) or choose to sample at later time points. Lastly, given that the experiment has been designed such that the *s_B_(t)* values do not exceed the sensitivity threshold, all that remains is to determine the *N_B_(t)* values using equation (7). Note that the CFU estimates are experimentally decoupled from *N_B_(t)* estimates. In our setup, the CFUs were determined from 1 ml of culture, while only the DNA extracted from 200 µl of culture was sequenced. Therefore, it is important to scale both measures to the same volume (see section 2.1). Finally, the microbial division and death rates are estimated by parameterizing equations 6ab with the CFU and the scaled *N_B_(t)* values.

To provide the reader with a guideline for the typical *m_I_* = *m_s_* = 2 experimental system (Fig. 2) we will assume a minimum number of sequences of 10^6^ = *I_2_ = S_2_* per sample and the sample size for the inoculum to be 10^6^ = *I_1_*. We choose a sampling time point *t* = 60 min and an initial concentration *N(0)* = 10^5^ cells per ml. The division rate and death rate are varied between [0, 0.046] min^−1^ and [0, 0.14] min^−1^ in 0.001 min^−1^ increments. The calculations of *s_B_(t)* using equation (8) shows that this system does not exceed the bottleneck sensitivity threshold for nearly the whole range for the division and death rate when at minimum 10^6^ = *S_1_* cells are sampled at *t* = 60 min. However, the bottleneck sensitivity does tend to sharply increase when the division rate and death rate approach 0 i.e. when *β* and *δ* are less than 0.005 min^−1^. Therefore, extra care should be taken in estimating the division and death rate for cells growing slowly, where the CFUs do not change appreciably during the time of observation.

One of the assumptions that underlies the RESTAMP model is that all cells divide and die at an equal rate (section 2). This is a limitation, which prevents using RESTAMP to study e.g. experiments with high selection pressure over extended periods of time, where mutants with altered fitness could accumulate or phenotypic heterogeneity, where genetically identical cells can manifest different phenotypes in a constant environment [28]. A striking example of this would be persister cells, where a fraction of cells in a genetically identical population survives longer in the presence of antibiotics [29]. In Figure Supplementary Fig. 6, we test the performance of the RESTAMP method in the presence of variation in the division rate and the death rate. The rates were independently drawn from a normal distribution where the mean death rate is 0.03 min^−1^ and the mean division rate is 0.01 min^−1^. We also consider the case where the rates are interchanged, i.e. the mean division rate is 0.03 min^−1^ and the mean death rate is 0.01 min^−1^. Figure Supplementary Fig. 6C shows the rate estimates for the former case while Figure Supplementary Fig. 6D shows the rate estimates for the latter case as functions of the standard deviation for the normal distribution, from which the rates were drawn. The results suggest that the estimated rates correspond to the mean rates for standard deviations smaller than 10^-3^ min^−1^, i.e. when the standard deviation relative to the mean is less than 10%. For wider distributions, when the standard deviation is 10^-2^ min^−1^, the rate estimates drift towards very large values. Accounting for this requires extending the RESTAMP theory for example by considering the division rate and death rate to be random variables. This potential extension of RESTAMP is best studied in the context of environments that induce phenotypic heterogeneity.

In this work, RESTAMP was tested and validated against experiments using *E. coli*. While the experimental (RE)STAMP protocol has been optimized and calibrated for bacteria, there is in principle no reason why it cannot be extended to other organisms. Likewise, the mathematical framework developed in this work does not make any organism-specific assumptions. We therefore believe that our approach can be useful to study the population dynamics of other pathogens, such as viruses.

## 5 Materials and methods

### 5.1 Emulating a death process by sampling

Here we seek to emulate a pure death process (Fig. 1A) by random sampling events (Fig. 1B) with the aim of devising an experiment where the death rate can be controlled. This control experiment can then be used to test how well the death rate, with the target division rate being 0, can be estimated using equations (6ab). The total average number of cells at time *t* in a pure death process with death rate *δ* is given by $<N\left(t\right)>=N\left(0\right)e^{-\delta t}$. This is emulated by sampling a small volume *Δv_t_ = Δv_0_e^-δt^* from a large volume. Since the rate estimates require not only the mean number of cells but also the mean founder population size at time *t*, one needs to ensure that <*N_B_(t)*> is correctly matched as well. Analogously, this translates to a requirement of matching the mean subpopulation size and the variance in the subpopulation size with the death process and the random sampling process. This is due to equation (3) that states the magnitude of the founder population size is inversely proportional to the variance in the subpopulation proportions. To proceed, we model the random sampling event as a multinomial random sampling process for which the probability of sampling *s* cells with a sequence tag insertion at site *i* is binomial.

(11) $$P\left(n_{i}=s\right)=\left(\begin{array}{c} N\left(t\right)\\ s \end{array}\right){f_{i}\left(0\right)}^{s}{\left(1-f_{i}\left(0\right)\right)}^{N\left(t\right)-s},s=0,1,\cdots ,N\left(t\right).$$

The binomial distribution applies for *n_i_(t)* cells surviving until time *t* in a death process as well where

(12) $$P\left(n_{i}\left(t\right)=s\right)=\left(\begin{array}{c} n_{i}\left(0\right)\\ s \end{array}\right)p^{s}{\left(1-p\right)}^{n_{i}\left(0\right)-s},s=0,1,\cdots ,n_{i}\left(0\right)$$

and *p = e^-δt^* is the probability of a single cell surviving until time *t*. The mean number of cells in the random sample is ${<n}_{i}>=<N\left(t\right)>f_{i}\left(0\right)=n_{i}\left(0\right)e^{-\delta t}=n_{i}\left(0\right)p$ which agrees with the mean number of cells for a death process. However, the variance in the number of cells with a sequence tag at site *i* in the sample *Δv_t_* is $Var\left(n_{i}\right)=n_{i}\left(0\right)p\left(1-f_{i}\left(0\right)\right)$ which is not equal to the variance in the death process where $Var\left(n_{i}\left(t\right)\right)=n_{i}\left(0\right)p\left(1-p\right)$. Hence, for the random sampling experiment to match a death process, *Var(n_i_)* need to be scaled with the factor (1-*p*)/(1-*f_i_(0)*). From equation (3), this translates to scaling the mean inverse founder population size as

(13) $$<{N_{B}\left(t\right)}^{-1}>\approx \frac{1}{k(1-p)}\sum_{i=1}^{k}\frac{\mathrm{Var}(f_{i}\left(t\right))}{f_{i}\left(0\right){\left(1-f_{i}\left(0\right)\right)}^{2}}$$

In our experiments *k* is 1000 and the factor (1-*f_i_(0)*) in the correction term is negligible and can be approximated as 1 with a negligible effect on the founder population size values. Thus, the experimentally determined mean founder population size as estimated in the random sampling experiment is simply scaled by the factor (1-*p*)^-1^.

### 5.2 Plasmid segregation

In this work, we adopt a simple mathematical framework for describing the dilution of an identifiable marker within cells that was recently used to quantify the dilution of self-aggregating fluorescent proteins [14]. Within this framework, the fraction of cells containing a plasmid at time *t*, <*F(t)>*, is given by < *F(t)>=F(0)e^-βt^* where *β* is the division rate and *r = β-δ* is the net growth rate. Solving for the rates we get

(14ab) $$\left\{\begin{array}{c} \beta =\frac{1}{t}ln\left(\frac{<F\left(0\right)>}{<F\left(t\right)>}\right)\\ \delta =\frac{1}{t}ln\left(\frac{<F\left(0\right)><N\left(0\right)>}{<F\left(t\right)><N\left(t\right)>}\right) \end{array}\right)$$

Where <*N(t)>* is the average total population size at time *t* and is experimentally estimated as the number of colony forming units.

### 5.3 Protocol for removing spurious sequence reads

The development of next generation sequencing technologies have revolutionized the quantification and analysis of the structures of microbial communities [9]. In particular, Illumina’s MiSeq platform [30] was successfully used in establishing the STAMP method for qualitatively investigating the population dynamics of cells [8]. The workflow of STAMP includes clustering and tallying individual sequence reads, the purpose of which is to remove spurious sequences that typically arise in using next generation sequencing technologies [31]. The aim of this section is to update the clustering step in the STAMP workflow [8] with a simple model for the expected number of extraneous spurious sequences. The consequence is that the sequence identity threshold in clustering is replaced with a query for the expected number of distinguishable subpopulations, i.e. the expected number of unique barcodes. The overarching strategy will be to estimate the expected number of the extraneous sequences that come about due to sequencing errors assuming that the error of a misread is equal and independent of basepair position. The additional sequences that remain after clustering *k* unique sequence tags including the ones that arise due to sequencing errors are then designated as spurious and removed from the analysis. All the variables used for modeling sequencing errors and their meaning are summarized in Table 2.

**Table 2:** A summary of the variables used in modeling sequencing errors.

Parameters	Meaning	Comments
*N_R_*	Random barcode sequence length.	In our experiments the length is *N_R_* = 30 bp.
*N_S_*	Strain barcode sequence length.	In our experiments the length is *N_S_* = 21 bp.
*k*	Number of unique barcodes/sequence tags.	In our experiments *k* = 1000.
*n*	Total number of sequences from the sequencing machine.	Typically on the order of 10^7^.
*n_F_*	Total number of sequences after filtering for the strain barcode.	Empirically it is often between 10^4-5^.
*p*	Probability of a correct nucleotide at any position in the sequence.	Typically, *p* is between 0.98 and 0.99 in our experiments.
*q*	Probability of an incorrect nucleotide at any position in the sequence.	Equal to 1-*p* and is typically between 0.01 (1%) and 0.02 (2%)
*m*	Number of mismatches.	
*P(m)*	Probability of m mismatches in the random barcode region.	
${\tilde{n}}_{m}$	Expected number of sequences containing *m* mismatches in the random barcode region.	
*m_max_*	The maximal number of mismatches for which the expected number of sequences are greater than one.	

Using STAMP, we generate on the order of 10^7^ 51 base pairs long sequence reads, where the first 30 base pairs are random and the last 21 base pairs are constant, and integrate them in a neutral position of the genome of our bacterial model. We denote the random sequence as the random barcode and the fixed sequence as the strain barcode and denote the lengths of these sequences as *N_R_* and *N_S_*, respectively. Artificial genetic variation is introduced in our bacterial model by using the random barcodes and is subsequently exploited to investigate the population dynamics of cells. The purpose of the strain barcode is to validate that the sequence reads with a random barcode are sequenced as opposed to sequencing random DNA snippets. In addition, the strain barcode allows for multiplexing different strains, which can be used to study interactions between populations. After filtering, the sequence reads with respect to the strain barcode and clustering the 100% matching sequences, our experiments result in 10^4^-10^5^ sequences. However, we expect *k* unique random barcodes where *k* is 1000 in this study. The additional sequences are due to a convolution of different effects such as sequencing errors, PCR errors or pooling of multiple barcodes [31]. The task at hand is to determine how many of the extraneous sequences arise due to sequencing errors and to designate the rest of the sequences as spurious which are then removed from the analysis. We use a simple model that assumes that the probability of reading an incorrect nucleotide at any position in the sequence is equal for all positions and independent of the position. Hence, the expected number of sequences after filtering with respect to the strain barcode (*n_F_*) is

(15) $$n_{F}=p^{N_{S}}N$$

where *p* is the probability of a correct nucleotide at any position and *N* is the total number of sequence reads. We use equation (15) to estimate *p* as

(16) $$p={\left(\frac{n_{F}}{N}\right)}^{1/N_{S}}$$

Given *p*, the probability of *m* incorrect reads in the barcode region is binomially distributed

(17) $$P\left(m\right)=\left(\begin{array}{c} N_{R}\\ m \end{array}\right)q^{m}{\left(1-q\right)}^{N_{R}-m}$$

where *q* = 1-*p* is the probability of an incorrect nucleotide at any position. After sorting the sequences with respect to abundance we remove all sequences beyond *k* with average number of *m* mismatches for which ${\tilde{n}}_{m}$ < 1 as spurious sequences. The average is used because we compare every extraneous sequence with all the *k* barcodes and thus we get a distribution of mismatches. This leaves *k* unique barcode sequences plus a mix of spurious- and sequencing error sequences. The last step in the algorithm is to pick out, top to bottom, $\left\{{\tilde{n}}_{0},{\tilde{n}}_{1},...,{\tilde{n}}_{\max}\right\}$ from the extraneous sequences and discard the rest as spurious sequences.

### 5.4 The variance in the proportion of cells with respect to repetitions for a birth–death process

Here we use the error propagation approximation to derive the variance in the proportion of subpopulation *i*, *Var(f_i_(t))*, for a stochastic birth–death process. Let *n_i_(t)* be the number of cells for subpopulation *i* at time *t* and let *N(t)* be the total number of cells at time *t*. Thus, *f_i_(t)* = *n_i_(t)*/*N(t)*. For notational simplicity we substitute *x* = *n_i_(t)*, *y* = *N(t)* and *f_i_(t)* = *g(x,y)* = *x*/*y*. Using the error propagation method, the variance of a ratio of random variables is

(18) $$\mathrm{Var}\left(g\left(x,y\right)\right)\approx {\left(\frac{\mu_{x}}{\mu_{y}}\right)}^{2}\left[\frac{\mathrm{Var}(x)}{\mu_{x}^{2}}+\frac{\mathrm{Var}(y)}{\mu_{y}^{2}}-2\frac{\mathrm{Cov}(x,y)}{\mu_{x}\mu_{y}}\right]$$

where *µ_x_*=<*n_i_(t)*>, *µ_y_*=<*N(t)* > and the angular brackets <> denote an average over repetitions. For independent subpopulations the covariance term reduces to *Var(x).*

(19) $$\mathrm{Cov}\left(x,y\right)=Cov\left(n_{i}\left(t\right),N\left(t\right)\right)=Cov\left(n_{i}\left(t\right),n_{i}\left(t\right)\right)=Var\left(n_{i}\left(t\right)\right)=Var\left(x\right)$$

For the birth–death process with division rate *β*, death rate *δ* and net growth rate *r* = *β*-*δ* we have the well-known relations for the means and the variances for the *i*:th subpopulation of cells and the total population.

(20abcd) $$\left\{\begin{array}{c} <n_{i}\left(t\right)>=n_{i}\left(0\right)e^{\mathrm{rt}}\\ <N\left(t\right)>=N\left(0\right)e^{\mathrm{rt}}\\ \mathrm{Var}\left(n_{i}\left(t\right)\right)=n_{i}\left(0\right)\frac{\beta +\delta }{\beta -\delta }e^{\mathrm{rt}}\left(e^{\mathrm{rt}}-1\right)\\ \mathrm{Var}\left(N\left(t\right)\right)=N\left(0\right)\frac{\beta +\delta }{\beta -\delta }e^{\mathrm{rt}}\left(e^{\mathrm{rt}}-1\right) \end{array}\right)$$

Substituting equations (19–20) in (18) the variance in the proportion of cells is

(21) $$\mathrm{Var}\left(f_{i}\left(t\right)\right)\approx \frac{\left(\beta +\delta \right)\left(1-e^{-rt}\right)}{\mathrm{rN}(0)}f_{i}\left(0\right)\left(1-f_{i}\left(0\right)\right)$$

### 5.5 Correcting for technical bottlenecks by the iterative application of the law of total expectation and the law of total variance.

In this section we consider a typical RESTAMP experiment, which involves technical bottlenecks such as sampling a small volume from a larger volume and sequencing due to the limited sequence chip capacity, in addition to a stochastic birth–death process. The variance of the founder population size will therefore include contributions due to these technical bottlenecks. An additional source of variation comes from estimating the initial tag frequencies, *f_i_(0)*, by experimental sampling. The task at hand is to separate these contributions from the birth–death process by propagating the added variance in the frequencies of sequence tags by iteratively applying the law of total expectation [23] and the law of total variance [23].

For simplicity and notational clarity, we consider a single technical bottleneck event. Using equation (1) to estimate the founder population size for cells having undergone a birth–death process and an additional downstream bottleneck event, we determine a founder population size that is a function of the total variance in the proportions, *Var(f_i,1_)* (equation (3)). See Fig. 2A for an illustration of the experimental setup. Using the law of total variance [23] *Var(f_i,1_)* can be decomposed as

(22) $$Var\left(f_{i,1}\right)=<Var\left(f_{i,1}|f_{i}\left(t\right)\right)>+Var\left(<f_{i,1}|f_{i}\left(t\right)>\right)$$

where <*Var(f_i,1_|f_i_(t))>* is the mean contribution to the total variance due to the bottleneck event. Here, *f_i,1_* is the frequency of sequence tag *i* after sampling the population of cells having undergone a random birth–death process for a time *t* and *f_i_(t)* is the frequency of sequence tag *i* at the end of the birth–death process at time *t* (Fig. 2A). By modeling the bottleneck as a multinomial random sampling process we have *<f_i,1_|f_i_(t)>=f_i_(t)*, i.e. sampling does not change the frequency of sequence tag *i* on average. Hence, *Var(<f_i,1_|f_i_(t) > ) = Var(f_i_(t))* which is the total variance due to a birth–death process. Applying the law of total variance on *Var(f_i_(t))* we get

(23) $$Var\left(f_{i}\left(t\right)\right)=<Var\left(f_{i}\left(t\right)|f_{i}\left(0\right)\right)>+Var\left({<f}_{i}\left(t\right)|f_{i}\left(0\right)>\right)$$

where <*Var(f_i_(t)|f_i_(0))>* is the mean variance in the sequence tag frequency due to birth–death process given an initial subpopulation proportion *f_i_(0)*. Substituting equation (23) in equation (22) and using *< f_i_(t)|f_i_(0)>≈f_i_(0)*, the total variance in the subpopulation proportions with a sequence tag *i* becomes

(24) $$Var\left(f_{i,1}\right)=<Var\left(f_{i}\left(t\right)|f_{i}\left(0\right)\right)>+<Var\left(f_{i,1}|f_{i}\left(t\right)\right)>+Var\left(f_{i}\left(0\right)\right)$$

Substituting equation (24) in equation (3), with *f_i_(0)* substituted for <*f_i_(0)*> as discussed in section 2.2, we get

(25) $$<{N_{B}\left(t\right)}^{-1}>\approx \frac{1}{k}\sum_{i=1}^{k}\frac{<Var\left(f_{i}\left(t\right)|f_{i}\left(0\right)\right)>}{{<f}_{i}\left(0\right)>\left(1-{<f}_{i}\left(0\right)>\right)}+\frac{1}{k}\sum_{i=1}^{k}\frac{<Var\left(f_{i}|f_{i}\left(t\right)\right)>}{{<f}_{i}\left(0\right)>\left(1-{<f}_{i}\left(0\right)>\right)}+\frac{1}{k}\sum_{i=1}^{k}\frac{Var\left(f_{i}\left(0\right)\right)}{{<f}_{i}\left(0\right)>\left(1-{<f}_{i}\left(0\right)>\right)}=<{N_{B}^{\mathrm{BD}}\left(t\right)}^{-1}>+<{N_{B}^{B}}^{-1}>+<{N_{B}^{0}}^{-1}>$$

where $<{N_{B}^{\mathrm{BD}}\left(t\right)}^{-1}>$ is the contribution to the founder population size due to a birth–death process, $<{N_{B}^{B}\left(t\right)}^{-1}>$ is the contribution due to the bottleneck (a multinomial random sampling event) and $<{N_{B}^{0}\left(t\right)}^{-1}>$ is the contribution due to sampling the inoculum at *t* = 0. In the context of sampling, the founder population size is equivalent to the sample size and we used equation (3) to equate the left hand side of equation (25) with the right hand side. We simplify the notation where *N_B_^B^* = *S_1_* and *N_B_^0^ = I_1_* and equation (25) becomes $<{N_{B}\left(t\right)}^{-1}>\approx <{N_{B}^{\mathrm{BD}}\left(t\right)}^{-1}>+<S_{1}^{-1}>+<I_{1}^{-1}>$. The founder population size due to a birth–death process, having accounted for a single bottleneck event, can therefore be estimated as

(26) $${<N}_{B}^{\mathrm{BD}}\left(t\right)>\approx \frac{1}{\frac{1}{k}\sum_{i=1}^{k}\frac{{\left(f_{i}\left(t\right)-{<f}_{i}\left(0\right)>\right)}^{2}}{{<f}_{i}\left(0\right)>\left(1-{<f}_{i}\left(0\right)>\right)}-<S_{1}^{-1}>-<I_{1}^{-1}>}$$

This result is generalized for *j* = 1, 2, 3…,*m_S_* bottleneck events for the sample taken at time *t* and *j* = 1, 2, 3…,*m_I_* bottleneck events for the reference sample at *t = 0* by iteratively applying the law of total variance, which leads to subtracting the sum of the average inverse sample sizes.

(27) $${<N}_{B}^{\mathrm{BD}}\left(t\right)>\approx \frac{1}{\frac{1}{k}\sum_{i=1}^{k}\frac{{\left(f_{i}\left(t\right)-{<f}_{i}\left(0\right)>\right)}^{2}}{{<f}_{i}\left(0\right)>\left(1-{<f}_{i}\left(0\right)>\right)}-\sum_{j=1}^{m_{s}}{<S}_{j}^{-1}>-\sum_{j=1}^{m_{I}}{<I}_{j}^{-1}>}$$

### 5.6 Strains

RESTAMP libraries of *E. coli* MG1655 (SoA2898) were constructed based on guidelines established in [8]. A chloramphenicol resistant gene (CmR) was PCR amplified from plasmid *pKD3* utilizing primers 5′-TCAGCGGCTACCGTGATTCATTCCCGCCAACAACCGCGCATTCCTCCAACGTGTAGGCTGGAGCTGCTTC-3′ and 5′-ATAAACTACAGCTGGCAGACAGCCGCTGCGAAGGCATTTTTGCACATGGCGCTCATTCCAGTCTACACGT-(N_30_)-ACTGGCCGTCGTTTTACAGCCATGGTCCATATGAATATCCTCCTTAG-3′, where N_30_ represents randomly integrated nucleotides, to create unique 30 bp tags. The PCR product was integrated into the genome of *E. coli* MG1655 between genes *codA* (b0337) and *cynR* (b0338) by the λ-red *pKD46* system per standard protocol [32]. A library of 1000 individual colonies, corresponding to ≤1000 unique tags, were individually grown to O.D._600_ 0.300, concentrated to O.D._600_ 10, combined with DMSO to 10% (v/v), aliquoted at 1 ml, and frozen at −80 °C. Each RESTAMP experimented utilized a frozen aliquot produced from the same stock. Plasmid *pAM34-pLac* was transformed into *E. coli* MG1655 by electroporation per New England Biolab’s protocol [12], [33]. Standard growth conditions for bacteria were LB-media (Miller, Sigma Cat. #L3147 or #L3522) at 37° C with broth cultures shaken at 225 rpm. When used, antibiotic concentrations were carbenicillin 50 µg/ml (Sigma, Cat. #C1389) and chloramphenicol 50 µg/ml (Sigma, Cat. #C0857). Isopropyl β-D-1-thiogalactopyranoside (IPTG) 1 mM was used for maintenance of *pAM34-pLac* replication in *E. coli* MG1655. Optical densities were measured at 600 nm (Thermo, Genesys20) in a 1 cm gap cuvette (Thermo, Cat. #5510).

### 5.7 Plasmid Segregation: Experimental

*E. coli* MG1655 *pAM34-pLAC* was recovered from frozen stocks on LB agar with carbenicillin 50 µg/ml and IPTG 1 mM. From a single colony 5 ml LB broth carbenicillin 50 µg/ml and IPTG 1 mM was seeded and grown for 16 h. Cultures were then pelleted and resuspended twice in 5 ml PBS (Sigma, Cat. #P4417) to remove residual IPTG. The culture was diluted 1:1600 into pre-warmed 37° C LB broth and grown to 1.0 O.D._600 nm_ to obtain ~ 20–30% *pAM34-pLac* positive cells. Culture was diluted to target starting concentration of 2.4x10^5^ CFU/ml in 25 ml of pre-warmed 37° C LB broth (~1:1470 dilution). For wild-type plasmid-loss controls, every 20 min, 600 µl sample was removed from the culture, serial diluted in PBS, and 100 µl of each dilution plated in triplicate for colony counts on LB agar carbenicillin 50 µg/ml and IPTG 1 mM and LB agar IPTG 1 mM. To simulate death, the starting culture was grown as above to a concentration of 2.4x10^5^ CFU/ml culture in 400 ml LB broth and then grown to an O.D._600_ 0.5. For a target death rate of 0.015 min^−1^, the culture was then serial diluted 4 times at 74 ml, 93 ml, 93 ml, and 93 ml to a total volume of 100 ml to represent targeted decreases in CFU at 20, 25, 30, and 35 min respectively. For a target death rate of 0.1 min^−1^, the sample was diluted 13.5 ml into 100 ml 4 fold for 20, 40, 60, and 80 mins. Immediately following dilutions, 100 µl were serial diluted and plated in triplicate on LB agar carbenicillin 50 µg/ml and IPTG 1 mM and LB agar IPTG 1 mM. Percent-positive *pAM34-pLac* colonies for were determined by the ratio of CFUs on carbenicillin containing agar to no antibiotics. All experiments were performed in triplicate.

### 5.8 RESTAMP

Frozen 1 ml aliquots of *E. coli* MG1655 (SoA2898) were recovered in 300 ml of pre-warmed 37° C LB broth and grown at 37° C to 0.3 O.D._600 nm_. Cells were then diluted in 25 ml of pre-warmed 37° C LB broth to a concentration of 2.4x10^5^ CFU/ml (~1:133 dilution). For measuring wild-type death rates, every 20 min 600 µl sample was removed from the culture, and serial diluted in PBS. 100 µl of each dilution was plated in triplicate for colony counts on LB agar chloramphenicol 5 µg/ml. Harvest plates for RESTAMP were prepared by plating 200 µl undiluted sample on to LB agar chloramphenicol 5 µg/ml and growing for 16 h at 37° C. To simulate death rates, 1 ml frozen aliquots of *E. coli* MG1655 (SoA2898) were recovered as described above and diluted to a calculated O.D._600 nm_ of 0.001 (~1x10^6^ CFU/ml). Sample volumes were then taken from the solution, with decreases in volumes representing increased time, and diluted to a volume of 1 ml in LB and allowed to grow to an O.D._600 nm_ of 0.1 (~1 h). Short log-phase growth does not alter tag frequencies. The samples were the pelleted, and DNA extracted for *N_B_* determination as described later. For the time points of 0, 20, 25, 30, 35, and 40 mins the volumes taken for the target death rate of 0.015 min^−1^ were 100, 74.1, 68.7, 63.8, 59.1, and 54.9 µl respectively. For the target death rate of 0.1 min^−1^, the sample volumes were 1000, 135.3, 82.1, 49.8, 30.2, and 18.3 µl. All experiments were performed in triplicate.

### 5.9 RESTAMP: Sample processing

Plates for RESTAMP analysis were harvested by placing 5 ml PBS on top of the plate and scraping. The O.D._600_ of a 1:10 dilution of the sample was measured and the dilution factor calculated for an O.D._600_ of 1.0. Using the calculated dilution factor the original solution was diluted and 1 ml pelleted. Genomic DNA extraction was performed on the pellet by adding 600 µl 2% sodium dodecyl sulfate (w/v) 0.5 M Ethylenediaminetetraacetic acid (aq) pH 8.0 lysis buffer for 5 min at 80 °C then 3 µl RNase A solution (Sigma, Cat. #R6148) was added for 30 mins at 37° C. Cell debris was precipitated with 200 µl 7.5 M ammonium acetate (aq) then centrifuged. DNA was precipitated from the supernatant with 800 µl of isopropanol then washed with 70% ethanol (aq) and suspended in 100 µl molecular grade water.

Illumina Miseq sequencing samples were generated using PCR with primers targeting the barcode flanking sequences with custom indexes and sequencing primer overhangs and manufacturer’s recommended P5 P7 regions (S1 Table). PCR was performed with OneTaq 2x Master Mix (NEB, Cat. #M0482) spiked with 1 U of Phusion High-Fidelity DNA Polymerase (NEB, Cat. #M0530). Three 50 µl PCR reactions were performed per sample with 20 cycle reaction to minimize replication bias then combined and purified using QIAquick PCR Purification Kit (Qiagen, Cat. # 28104) per manufacturer’s protocol. PCR products were confirmed by gel electrophoresis and concentration determined by Nanodrop (ThermoFisher, ND-1000). Samples were combined for a concentration of 10 ng/µl of each sample. The final concentration of the sample was measured by Qbit (ThermoFisher, Cat. #Q32854) and diluted to 8 nM. Sequencing was performed on Illumina MiSeq System TruSeq HT assay per manufacturer’s protocol using MiSeq Reagents Kit v2 50 cycles (Illumina, Cat. #MS-102–2001) with custom sequencing primers (S1 Table).

## 6 Code

Codes for all figures were implemented in MATLAB (R2017b, The MathWorks, Natick, MA, USA). All code for reproducing the results and the raw sequencing data is available on SourceForge: https://sourceforge.net/projects/restamp/. All scripts are free software and are free to redistribute and/or modify under the terms of the GNU General Public License as published by the Free Software Foundation, either version 3 of the License, or any later version.

To make the method in this work accessible we implemented an analysis pipeline in Matlab R2017b that takes next-generation sequencing files and produce founder population size values via a graphical user interface. The analysis pipeline is an extension of [8] and has been updated with a protocol for removing extraneous spurious sequences that typically arise in next-generation sequencing technologies [31] (see *5.3 – Protocol for removing spurious sequence reads*). This software was used to analyze the next-generation sequencing data and produce founder population size values, which were subsequently used to calculate the rates using equations (6ab). The software is freely available for download on https://sourceforge.net/projects/restamp/.

## CRediT authorship contribution statement

**Anel Mahmutovic:** Conceptualization, Data curation, Formal analysis, Investigation, Methodology, Software, Supervision, Validation, Visualization, Writing - original draft, Writing - review & editing. **Aaron Gillman:** Data curation, Investigation, Validation, Visualization, Writing - original draft, Writing - review & editing. **Silje Lauksund:** Investigation, Methodology, Supervision. **Natasha-Anne Robson Moe:** Data curation, Investigation. **Aime Manzi:** Data curation, Investigation. **Merete Storflor:** Investigation, Supervision. **Pia Abel zur Wiesch:** Conceptualization, Funding acquisition, Investigation, Resources, Supervision, Writing - review & editing. **Sören Abel:** Conceptualization, Funding acquisition, Investigation, Project administration, Resources, Supervision, Visualization, Writing - review & editing.

## Declaration of Competing Interest

The authors declare that they have no known competing financial interests or personal relationships that could have appeared to influence the work reported in this paper.

## Figures and Tables

**Supplementary Fig. 6:**